# Sources of variability in SERS spectra of bacteria: comprehensive analysis of interactions between selected bacteria and plasmonic nanostructures

**DOI:** 10.1007/s00216-019-01609-4

**Published:** 2019-03-04

**Authors:** Evelin Witkowska, Krzysztof Niciński, Dorota Korsak, Tomasz Szymborski, Agnieszka Kamińska

**Affiliations:** 1 Institute of Physical Chemistry, Polish Academy of Sciences, Kasprzaka 44/52, 01-224 Warsaw, Poland; 2 Faculty of Biology, Department of Applied Microbiology, Institute of Microbiology, University of Warsaw, Miecznikowa 1, 02-096 Warsaw, Poland

**Keywords:** Surface-enhanced Raman spectroscopy (SERS), Wire mesh, SERS platform, *Escherichia coli*, *Bacillus subtilis*

## Abstract

The surface-enhanced Raman spectroscopy (SERS)-based analysis of bacteria suffers from the lack of a standard SERS detection protocol (type of substrates, excitation frequencies, and sampling methodologies) that could be employed throughout laboratories to produce repeatable and valuable spectral information. In this work, we have examined several factors influencing the spectrum and signal enhancement during SERS studies conducted on both Gram-negative and Gram-positive bacterial species: *Escherichia coli* and *Bacillus subtilis*, respectively. These factors can be grouped into those which are related to the structure and types of plasmonic systems used during SERS measurements and those that are associated with the culturing conditions, types of culture media, and method of biological sample preparation.

**Electronic supplementary material:**

The online version of this article (10.1007/s00216-019-01609-4) contains supplementary material, which is available to authorized users.

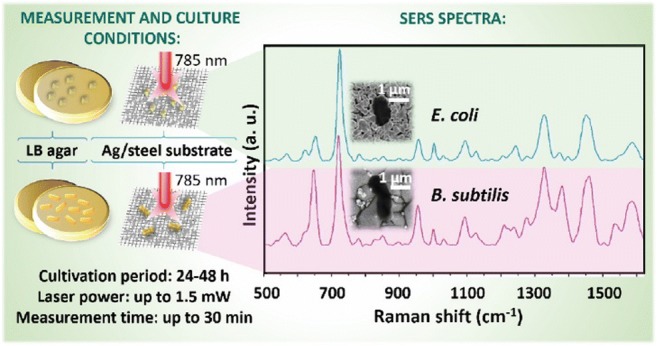

## Introduction

Surface-enhanced Raman spectroscopy (SERS) spectroscopy is a technique based on the study of oscillations of molecules located on roughened metal surface. The amplification of the SERS signals occurs via the formation of charge transfer complex between the analyte and the SERS substrate, but mainly through the electromagnetic interaction of light with metal, which produces large amplifications of the laser field. The electromagnetic enhancement is connected with the presence of surface plasmons—coherent delocalized electron oscillations across the surface [1].

High selectivity, sensitivity, and the possibility to analyze a trace amount of substances are the main advantages proposed by SERS. In addition, there is no need to use expensive reagents and labels or to employ qualified personnel to carry out the experiment. The SERS spectrum of chemical compounds resembles the normal Raman spectrum (they are both the source of information about the structure of studied compounds). Unfortunately, in the case of biological samples, the background fluorescence often completely covers signals from the sample during normal Raman measurements and, therefore, it is very difficult to obtain pure Raman spectrum.

The power of SERS lies also in its ability to identify chemical species in a very fast way. It can be used to detect and identify pigments [2], poisons [3], narcotic substances [4–6], and materials of biological origin [7–10]. It is also applied in examination of single molecules [11, 12] and in identification of structures more complicated than chemical compounds, namely viruses [13, 14], fungi [15], cancer cells [16–18], and bacteria [19–21].

The topic of microorganism identification by SERS is hugely important from biological, biotechnological, and medical points of view. In these branches of science, the new methods of bacterial identification are extremely desired. This is due to the fact that currently used techniques, including polymerase chain reaction, biochemical tests, and immunochemical methods, despite their high accuracy, still cannot overcome the main factor which makes them imperfect—time consumption. The SERS technique may resolve this problem, as the single measurement can be done in only few seconds. For the abovementioned reasons, over the last years, the interest in SERS of bacteria is big. This is revealed by an increase in the number of scientific publications on this topic (see Electronic Supplementary Material (ESM) Fig. S1).

Despite the knowledge about the principles of SERS mechanism, the detailed information remains still unknown. The more complex the examined sample, the more it is difficult to determine the origin of the vibrations observed in the SERS spectrum. Therefore, spectral analysis of bacteria, which are composed of hundreds of different molecules, is a serious issue. SERS technique has also some restrictions. First, the analyte should be very pure as the contamination of the sample may result in additional bands in the spectrum. Moreover, the SERS effect is limited to ~ 20 Å from the surface of the metal substrate, so in the case of measurements of viable bacterial cells, the obtained signals may be assigned only to first few layers of the cell (cell wall, cell membrane, and molecules attached to them).

This work is the first step to standardize the conditions, which enable to obtain reproducible SERS spectra of particular bacteria. The factors influencing the spectrum and signal enhancement of selected bacterial species in SERS have been studied. They can be grouped into those which are related directly to the SERS platforms and measurement parameters and those which are associated with the cell cultivation process and method of sample preparation.

Not all SERS substrates are suitable for bacterial cell measurements. In some cases, spectra cannot be compared with each other, especially if they were obtained by using different SERS substrates covered with distinct SERS-active metals. Equally important for the results are measurement parameters: time of exposure to laser radiation, laser line, and laser power. It is important to verify the influence of the type of plasmonic structures on bacterial SERS spectra. Here, we show, for the first time, how the type of used SERS substrates (obtained in different production processes), the duration of cell cultivation, and additional treatment during sample preparation affect SERS spectra of bacteria. Moreover, during SERS measurements, we have used four different laser lines (325 nm, 514 nm, 633 nm, and 785 nm) and six laser power levels of the selected laser line. We have also checked the impact of SERS-active metal/alloy and the duration of the experiment on the SERS spectra of bacteria as silver nanoparticles (NPs)/nanostructures are toxic to bacteria. This toxic effect is connected with generation of reactive oxygen species (ROS) upon exposure of cells to silver (Ag) NPs [22]. As the result, they may indirectly disrupt the function of cytoplasmic membrane, inactivate key enzymes, interact with nucleic acids, interfere with metabolic and respiratory processes, and cause death of the cell [23]. This, in turn, may lead to some changes in the quality of obtained SERS spectrum.

We present as well how the culture conditions may affect the structure and chemical composition of bacteria and, thus, their SERS spectra. Precisely, we show how the type of culture media and death of bacteria may affect their SERS signal intensity and quality. We have focused on two model bacterial species: Gram-negative *Escherichia coli* and Gram-positive *Bacillus subtilis*.

## Materials and methods

### Bacterial species

Two bacterial strains (*E. coli* K12 and *B. subtilis* 168) were obtained from the Department of Applied Microbiology, University of Warsaw, Poland.

### Culture conditions and sample preparation

#### Storage

Cultures of *E. coli* and *B. subtilis* were maintained in Trypticase soy–yeast extract agar (TSYEA) (Oxoid, Basingstoke, Hampshire, UK) at 4 °C throughout the study period and stored at − 80 °C in brain heart infusion (BHI) broth supplemented with 20% glycerol.

#### Culture media and conditions of bacterial cultivation

The following culture media were purchased from Biocorp, Poland: Luria–Bertani (LB) agar medium, BHI agar, and Mannitol Egg Yolk Polymyxin (MEYP) agar for cultivation of *B. subtilis* and LB agar medium, BHI agar, Tryptone Bile X-Glucuronide (TBX) agar, and Chromogenic Coliform Agar (CCA) for cultivation of *E. coli*. All the plates were incubated at 37 °C for 24 h. Additionally, the plates with LB agar and *E. coli* or *B. subtilis* were cultured for 48 h and 72 h to check the influence of the culture duration on the SERS spectrum.

#### Death of bacteria

In order to cause death of bacterial cells, they were suspended in 70% ethanol, frozen in saline solution at − 80 °C for 24 h, centrifuged (13,150×*g*) for 10 min, heated to 100 °C in saline solution for 1 h, and exposed to UV light for 1 h.

#### Sample preparation for SERS measurements

For viable *E. coli* and *B. subtilis* cells, after 24 h (or 24 h, 48 h, and 72 h in the case of bacteria cultured on LB agar medium), three single colonies were placed via a sterile plastic inoculating loop into 200 μL of sterile 0.9% NaCl solution, mixed, and centrifuged for 3 min at 1070×*g*. The centrifugation process in the saline solution was repeated three times to obtain a solution of clean bacterial cells. Purified bacteria were finally suspended in 10 μL of sterile 0.9% NaCl solution. The mixtures were next placed over the SERS substrates and measured after ~ 5 min (or 5 min, 15 min, and 45 min in the case of bacteria cultured on LB agar medium) with a Raman spectrometer.

Previously purified cells of *E. coli* and *B. subtilis*, which were used in experiments showing the influence of bacterial death to their SERS spectrum, were suspended in 10 μL of sterile 0.9% NaCl solution. Subsequently, they were immediately exposed to specific factor causing death (70% ethanol, − 80 °C, and centrifugation at 13,150×*g*, 100 °C, UV light). Next, the liquid was placed over the SERS substrate and measured after ~ 5 min with a Raman spectrometer.

#### Sample preparation for Raman measurements

After 24 h of bacterial cultivation on LB agar medium, about ten single colonies were placed via a sterile plastic inoculating loop into 200 μL of sterile 0.9% NaCl solution, mixed, and centrifuged for 3 min at 1070×*g*. The centrifugation process in the saline solution was repeated three times to obtain a solution of clean bacterial cells. Purified bacteria were finally suspended in 10 μL of sterile 0.9% NaCl solution. The mixtures were next placed over the microscopic slides, left for ~ 5 min to dry out, and measured with a Raman spectrometer.

### Instrumentation

#### Raman and SERS spectroscopy

Measurements were carried out with a Renishaw inVia Raman system equipped with a 300-mW diode laser–emitting 785-nm line, a 50-mW He-Ne laser–emitting 633-nm line, a 50-mW diode laser–emitting 532-nm line, and a 200-mW He-Cd laser–emitting 325-nm line. These lines were used as an excitation source (*λ*_ex_). The light from the lasers was passed through a line filter and focused on a sample mounted on an X–Y–Z translation stage with a × 20 microscope objective (NA = 0.25). The beam diameter was approximately 5 μm. The laser power at the sample was 1.5 mW, 1 mW, 1.2 mW, and 1.6 mW, respectively, for 785-nm, 633-nm, 532-nm, and 325-nm laser lines. For the 785-nm laser line, the power levels 0.08 mW, 0.16 mW, 0.8 mW, 1.5 mW, 8.0 mW, and 16.0 mW were also tested. The microscope was equipped with three different diffraction gratings (1200 grooves/mm [for the 785-nm line], 1800 grooves/mm [for the 633-nm and 532-nm lines], and 2400 grooves/mm [for the 325-nm line]), cutoff optical filters, and a 1024 × 256-pixel Peltier-cooled RenCam CCD detector, which allowed registering the Stokes part of Raman spectra with 5–6 cm^−1^ spectral resolution and 2 cm^−1^ wavenumber accuracy. The experiments were performed at ambient conditions using a back-scattering geometry.

#### Scanning electron microscopy

Observations were performed under high vacuum using the FEI Nova NanoSEM 450. The accelerating voltage was in a range from 2 kV up to 10 kV. The wire mesh samples with bacterial species were observed without any additional layer of gold.

### SERS substrate preparation

#### Ag disc

Silver SERS substrates were obtained by polishing discs (*Ø* = 10 mm, *H* = 5 mm) with Al_2_O_3_ slurries in the SII OFL-12 Fiber Polisher (Seiko Instruments). First, slurry contained particles with a size of 0.5 mm, whereas the second one, particles with a size of 0.3 mm. The polishing process was continued until obtaining a mirrored surface of the disc. Next, the discs were washed and sonicated in ultrasonic bath for 10 min in 70% ethanol solution. The sonication process was repeated in Millipore water. Subsequently, the discs were electrochemically roughened by oxidation/reduction cycles (ORCs) in the electrochemical cell filled with 0.1 M KCl solution. The three ORCs were applied (0.5 V and − 0.5, both for 40 s; 0.5 V and − 0.5, both for 15 s; 0.5 V for 15 s and − 0.5 for 30 s). In the last step, the reduction potential of − 0.4 V was applied for 300 s. Finally, the silver discs were rinsed with Millipore water, dried, and used immediately in SERS experiments. The scanning electron microscopy (SEM) images of the surface of Ag disc are presented in ESM Fig. S2a and in the section “The influence of base material building the SERS substrate.”

#### Silver-coated silicon plate (Ag/Si)

One-sidedly polished silicone plates (*Ø* = 25 mm) were placed in a beaker filled with 30% KOH solution for 40 min at 50 °C. Next, after rinsing the plates in Millipore water, they were sonicated in ultrasonic bath for 15 min at 50 °C in the following substances: acetone, isopropyl alcohol, and Millipore water. Next, the platforms were dried for 30 min at 50 °C and placed in a physical vapor deposition (PVD) device and sputtered with a 10-nm layer of silver. The substrates were used immediately after their preparation. The SEM images of the surface of Ag/Si substrate are presented in ESM Fig. S2b and in the section “The influence of base material building the SERS substrate.”

#### Silver-coated steel mesh (Ag/steel)

In order to obtain a Ag/steel SERS substrate, a wire mesh sample (40 mm × 40 mm) was placed in a beaker filled with acetone and sonicated for 10 min in ultrasonic bath at a temperature of 50 °C. The process was repeated three times: with a new portion of acetone at 50 °C, with isopropyl alcohol at 50 °C, and with Millipore water at ambient temperature. Next, the cleaned wire mesh was dried for 30 min at 50 °C, placed in a PVD device, and sputtered with a 50-nm layer of Ag (or of Au, Cu, or Ag/Au alloy). The substrates were used immediately after their preparation. The SEM images of the surface of Ag/steel substrate are presented in ESM Fig. S2c and in the section “The influence of base material building the SERS substrate.”

### Collection of the SERS and Raman spectra

The Raman and SERS spectra of bacterial cells were recorded in mapping mode almost immediately after placing the microscope slide or SERS platform under the microscope lens. Before performing SERS measurements, Raman spectra of pristine SERS substrates were recorded (ESM Fig. S3).

For each sample, 30 spectra were collected; thus, each spectrum presented in this publication is a result of averaged 30 measurements. For all spectra, the laser power was set to ~ 1.5 mW. Next, the spectra were saved and processed with OPUS software, ver. 2012 (Bruker Optik GmbH, Germany). The mentioned program allowed to (i) average the spectra within one map, (ii) remove the background due to baseline correction (selected method: concave rubberband correction; number of interactions = 10), (iii) smooth the final spectrum (Savitzky-Golay filter, 9 points), and (iv) normalize the spectra using min–max normalization; normalization was not applied when differences in absolute spectral intensity among different conditions were evaluated. This applies to the spectra depicted in Fig. 6 and ESM Figs. S7 and S9. The abovementioned bands are crucial for bacterial identification in SERS technique, and the normalization would blur differences in the intensity observed for these bands. The time required for completing a single SERS spectrum was 12 s, whereas for completing a single Raman spectrum, it was 5 min.

### Experimental conditions

The scheme of all experimental conditions is depicted in ESM Fig. S4. In the present work, the following eight experimental conditions (connected with bacterial cultivation, sample preparation, or SERS measurement) were tested:

- Diverse base materials for preparation of SERS substrates (steel mesh, bulk silver, and silicon; ESM Fig. S4a)
- The presence and absence of the SERS substrate (normal Raman vs SERS spectra; ESM Fig. S4b)
- Different SERS-active metals (Ag, Au, Ag/Au alloy, and Cu; ESM Fig. S4c)
- Different laser lines (532 nm, 633 nm, and 785 nm) and laser power levels of 785-nm line (0.08 mW, 0.16 mW, 0.8 mW, 1.5 mW, 8.0 mW, and 16.0 mW; ESM Fig. S4d)
- Diverse culture media (LB agar, BHI agar, and MEYP agar for cultivation of *B. subtilis* or LB agar, BHI agar, TBX agar, and CCA for cultivation of *E. coli*; ESM Fig. S4e)
- Duration of bacterial cultivation (24 h, 48 h, or 72 h; ESM Fig. S4f)
- Influence of bacterial death on their SERS spectra (factors causing cell damage: 70% ethanol, − 80 °C, centrifugation at 13,150×*g*, 100 °C, UV light; ESM Fig. S4g)
- Time spent by bacterial sample on SERS substrate (5 min, 15 min, and 45 min; ESM Fig. S4h).

## Results and discussion

Although numerous reports about Raman and SERS spectroscopy for bacterial imaging are available, there was no attempt to create a uniform SERS protocol for bacterial detection. Therefore, obtaining the similar SERS spectra of particular bacterial species is still a challenging task. The optimization of the conditions and factors influencing SERS, which will lead to the development of uniform, reproducible SERS spectra of bacterial cells, is needed. As SERS is a very sensitive spectroscopic technique, the enhancement of Raman signal should depend on many factors, including the type of SERS-active metal, the frequency of incident light, effective Raman cross section, and the structure of the SERS substrate. The size of metal nanostructures used to produce a SERS substrate is also a crucial issue. The huge differences between the spectra may also result from the different cell culture conditions and measurement parameters. This leads to many ambiguities and to the inability to compare obtained spectra. Such dissimilar spectral images hamper the practical application of SERS-based bacterial identification in many fields of science.

### Normal Raman versus SERS

As mentioned before, although the SERS spectrum resembles the normal Raman spectrum, especially in the case of simple chemical compounds, this is not the rule for complex biological substances and biomolecules. In ESM Fig. S5, the normal Raman spectra of *E. coli* and *B. subtilis* are compared with their SERS spectra. In can be noticed that the SERS spectra of both bacterial species are of extremely better quality than normal Raman spectra. This is connected with the fact that in normal Raman, the high background in the spectrum, which is due to the fluorescence and the emission continuum, completely covers the signal from bacterial cells. This problem is eliminated in SERS experiment, to significant extend, when using appropriate SERS substrates, intended for measurements of biological samples. Another argument behind the growing use of SERS is the fact that in this technique, the trace amounts of the chemical or biological compounds allow to obtain the signal of high quality. Obviously, the sample should be first properly prepared (e.g., filtered and rinsed several times) to avoid the adsorption of unwanted sample components to the SERS substrate. Additionally, the duration of SERS experiment for biological samples is considerably short—in order to obtain a single SERS spectrum of bacteria, the time of 12 s was sufficient.

In conclusion, SERS allows the detection of very low concentration levels of microorganisms in the sample. It also provides the differentiation and identification of very similar species and strains. The detection of such bacterial cells is ultrasensitive and rapid and requires no extrinsic labeling steps. The huge signal from SERS enhancements, coupled with, i.e., large scattering cross sections of microbial cells, allows to obtain spectra from individual bacterial cells. Moreover, the surface of a bacterial cell consists of a diverse range of biomolecules, almost each of them giving a characteristic Raman spectrum. The mixture of these molecules constitutes the total, unique bacterial SERS signal.

### The influence of base material building the SERS substrate

**Fig. 1:**
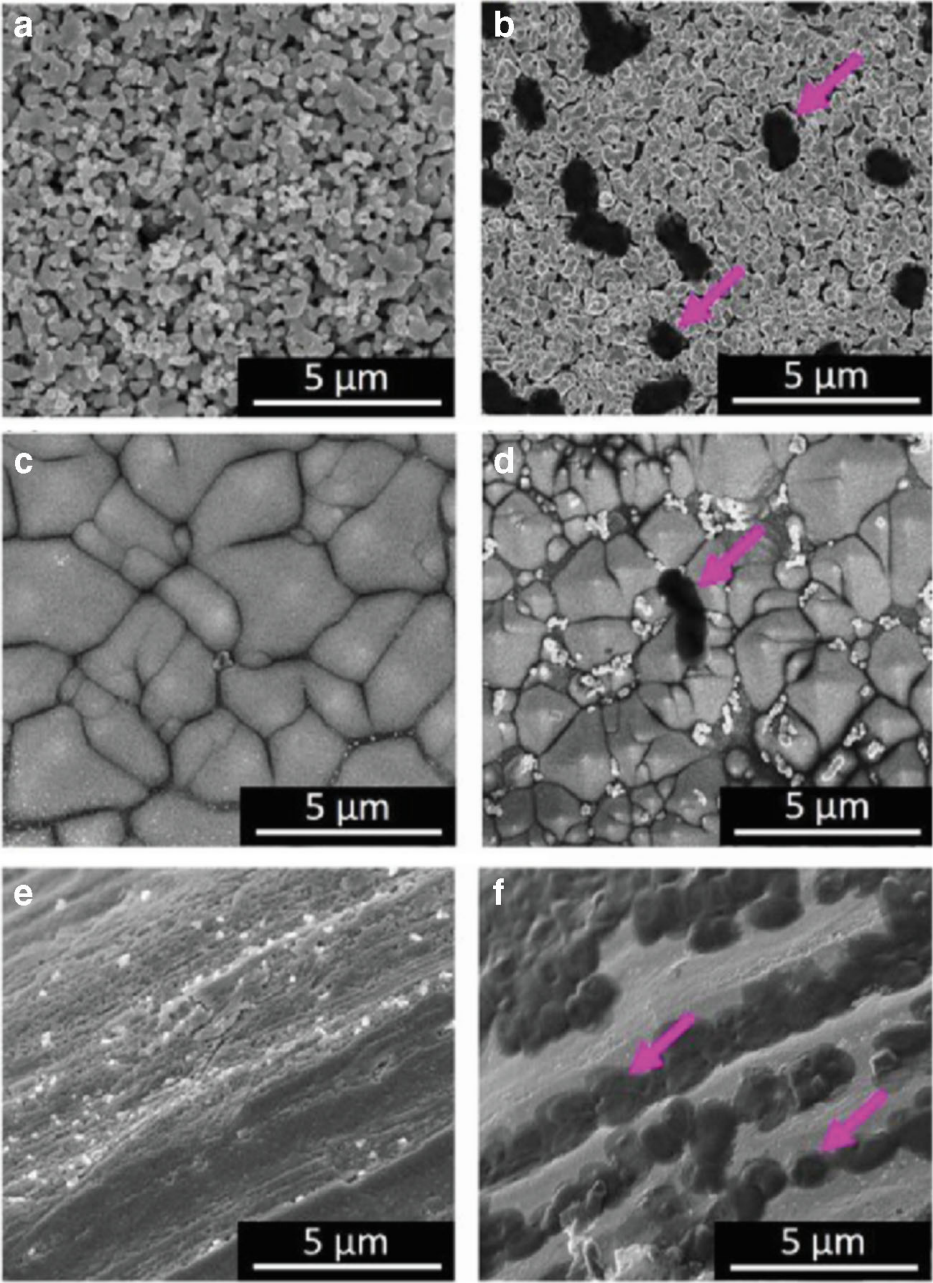
SEM images of different SERS substrates and bacterial cells adsorbed to them: Ag disc (**a**) without and (**b**) with *E. coli* cells, chemically etched silicon covered with Ag nanostructures (**c**) without and (**d**) with *B. subtilis* cell, and steel mesh covered with Ag nanostructures (**e**) without and (**f**) with *E. coli* cells. Pink arrows indicate bacterial cells

There are a lot of patents and publications describing different SERS substrates and methods of their production [19, 24–37]. However, not all of the SERS substrates give satisfactory enhancement of Raman signal, especially while measuring biological substances. Here, we performed SERS measurements on different SERS substrates obtained by (i) cyclic voltammetry with a silver disc as a working electrode, (ii) sputtering silver via PVD on chemically etched silicon, and (iii) sputtering silver via PVD on steel mesh (see Fig. 1). The aim of this experiment was to compare substrates with different roughness. Their description and additional SEM images can be found in ESM Fig. S2.

**Fig. 2:**
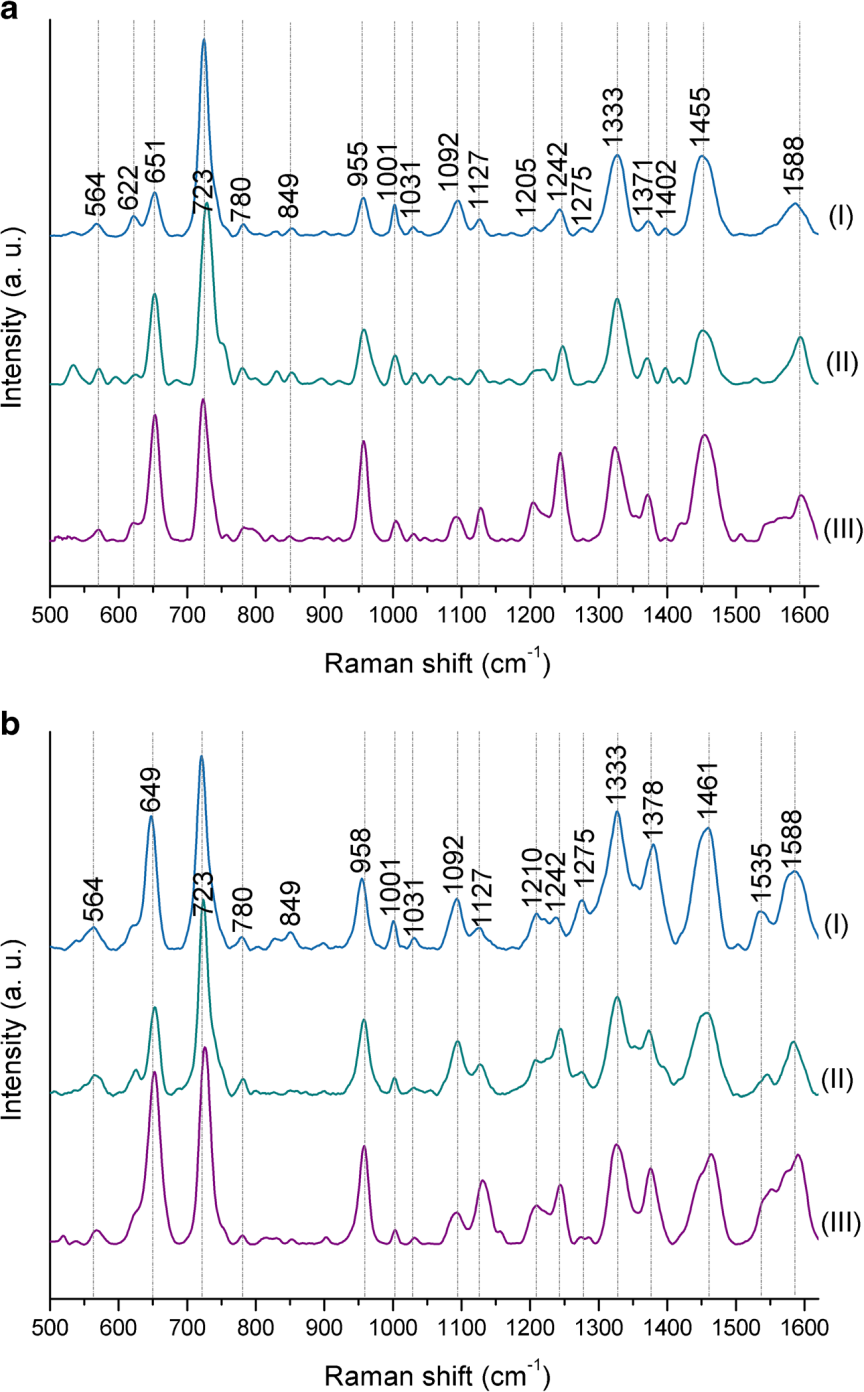
The averaged SERS spectra of (**a**) *E. coli* and (**b**) *B. subtilis* measured on (**I**) Ag/steel, (**II**) Ag disc, and (**III**) Ag/Si SERS substrates. Bacteria were cultured on LB agar medium (24 h, 37 °C). All presented spectra were averaged from 30 SERS measurements performed with the 785-nm laser line (1.5 mW), baseline corrected, smoothed, and normalized

As one can observe in Fig. 2, the SERS spectra obtained with the use of mentioned SERS platforms are very similar within bacterial species (*E. coli* or *B. subtilis*)—the locations and intensities of the main bands remain unchanged regardless of the type of used SERS platform. In each SERS spectrum, for both *E. coli* and *B. subtilis*, it is possible to observe bands at around 565 cm^−1^ (C–S–S–C), 620 cm^−1^ (phenylalanine), 720 cm^−1^ (adenine in FAD and NAD), 955 cm^−1^ (C=C deformation), 1000 cm^−1^ (phenylalanine), 1090 cm^−1^ (C–C skeletal and C–O–C stretching from glycosidic link), 1240 cm^−1^ (amide III), 1330 cm^−1^ (CH_2_/CH_3_ wagging mode in purine bases of nucleic acids), 1450 cm^−1^ (CH_2_/CH_3_ deformation of proteins and lipids), and 1590 cm^−1^ (phenylalanine, hydroxyproline, tyrosine, etc.) [7].

In conclusion, the method and the type of base material used during SERS substrate production should not result in obtaining totally different SERS spectra of the same bacterial species. The minor differences are probably connected with the size of SERS-active nanostructures and, thus, with the properties of hot spots which can be described as the regions of very strong local field enhancement caused by the surface plasmon resonance. The presence of strong additional bands or absence of the main spectroscopic features of bacteria is a key problem that appears during comparison of the SERS spectra of the same bacterial species presented in different scientific publications, e.g., *E. coli* [38–44]. This may be connected with the purity of the chemical/physical method used to produce SERS platform. As long as the method does not use numerous chemical compounds which may contaminate SERS substrate, e.g., carbonaceous contamination [45], the obtained SERS spectra of bacterial cells should not change dramatically. The visible changes in relative intensities of the same bands are most probably related with the effect of molecular orientation in relation to the polarization of plasmon excitations in the metal substrate. Of course, other factors, including cell culture conditions and the method of sample preparation, may affect the SERS spectra, but these issues will be discussed later in this work.

### The influence of the SERS-active metal used to produce a SERS substrate

It should be highlighted that taking into account the size of whole pathogenic cells and the distance dependence in SERS effect [46], there is consensus among researchers that the observed bacterial spectroscopic features are dominated mainly by their metabolites and outer cell wall constituents such as membrane proteins, amino acids, lipopolysaccharides, fatty acids, and derivatives of purines [47–49].

In order to observe a SERS signal, the adsorption of the analyzed sample onto SERS substrate is required. The type, shape, and size of nanostructures building the platform affect the surface plasmon resonance giving higher or lower Raman signal enhancement. Moreover, the intensity of SERS signal will be different for small molecules than for single cells composed of thousands of molecules. It is because that a whole cell is too large to occupy a hot spot on the substrate. Hot spots are usually localized in the crevices of the metal nanostructures, and their width should not exceed ~ 2 nm. Such small gaps can be settled only by single molecules and not by relatively large cells (with the diameter of ca. 1–2 μm). Therefore, the enhancement factors for these two types of samples would be different.

Although the SERS effect was observed for metals, such as Li, Na, K, Rb, Cs, Al, Ga, In, Pt, Ag, Au, Cu, and Rh, and their alloys [50], the strongest SERS enhancements are obtained for Ag, Au, and Cu [51]. For this reason, the SERS measurement of bacteria was performed on four types of SERS substrates composed of steel microwires covered with Ag, Au, Ag-Au alloy, and Cu nanostructures. All types of metal-coated steel SERS platforms were prepared in the same way (50 nm of appropriate metal was sputtered onto stainless steel wire mesh via PVD and placed in sterile Petri dishes), and therefore, their morphology was expected to be comparable.

**Fig. 3:**
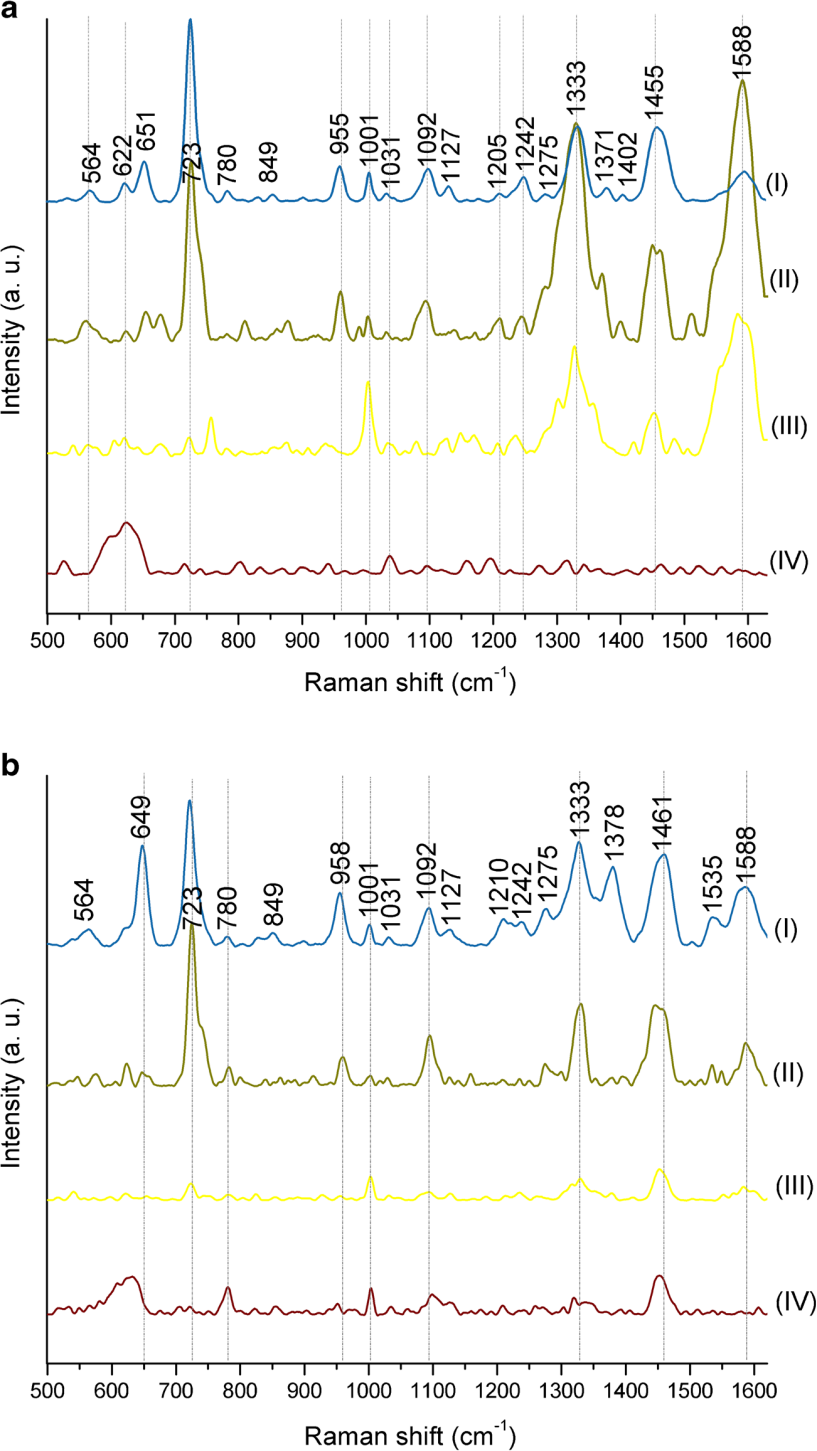
The SERS spectra of (**a**) *E. coli* and (**b**) *B. subtilis* measured on steel mesh SERS substrates covered with 50 nm of (**I**) Ag, (**II**) Ag/Au alloy, (**III**) Au, and (**IV**) Cu. Bacteria were cultured on LB agar medium (24 h, 37 °C). All presented spectra were averaged from 30 SERS measurements performed with the 785-nm laser line (1.5 mW), baseline corrected, smoothed, and normalized

As shown in Fig. 3, the highest signal-to-noise ratio (SNR), the most intense SERS bands and therefore, the most characteristic SERS spectra are observed for Ag/steel and Au/Ag/steel platforms, both for *E. coli* and *B. subtilis* (Fig. 3a-b, I and II).

The Au/steel platform also shows some characteristic bands, especially at around 1000 cm^−1^, 1330 cm^−1^, 1450 cm^−1^, and 1590 cm^−1^ (Fig. 3a, b (III)), but other bands typical for bacterial cells, e.g., at around 620 cm^−1^, 650 cm^−1^, 730 cm^−1^, 960 cm^−1^, and 1100 cm^−1^ are absent or hardly noticeable. Additionally, the intensities of observed bands are very low in comparison to Ag and Ag-Au nanostructures. The Cu substrate, on the contrary, showed only one band, at around 620 cm^−1^, originating from bacterial cell components of *E. coli* (Fig. 3a (IV)), or showed only four bands at ca. 620 cm^−1^, 780 cm^−1^, 1000 cm^−1^, and 1450 cm^−1^ in the case of *B. subtilis* (Fig. 3b (IV)). As can be concluded, after the comparison of SERS spectra from all considered substrates, the platforms containing silver give the most characteristic and are rich in bands for bacterial spectra.

For further experiments, the substrates consisting of steel mesh covered with Ag nanostructures were chosen. The main reason of selecting this type of SERS substrate was the possibility of observing the biggest difference between *E. coli* and *B. subtilis* SERS spectra. The second reason is connected with the fact that the Ag/steel platforms are cheaper to produce than the Ag/Au/steel platforms, and therefore, they are affordable for many laboratories.

### The influence of the laser line wavelength and power

Several research groups have investigated the SERS spectra of both Gram-positive and Gram-negative bacteria at different wavelengths (for more information, see ESM, section 6). Based on the literature, it can be concluded that in SERS analysis of bacteria, the 633-nm and 785-nm lasers are the most extensively used laser lines. These excitation sources have relatively low photon energy and low fluorescence background and do not cause the photodamage of samples.

The wavelength of the excitation laser and its power on the irradiated sample (power/area) are the crucial factors that affect the high spectral resolution. The spatial resolution is defined by the optics of microscope objective and the wavelength of the laser. In biological studies, the near-infrared (NIR) lasers at 785 nm and 830 nm, which have relatively low photon energy and enable the reduction of fluorescence contribution in Raman spectrum, are commonly used.

**Fig. 4:**
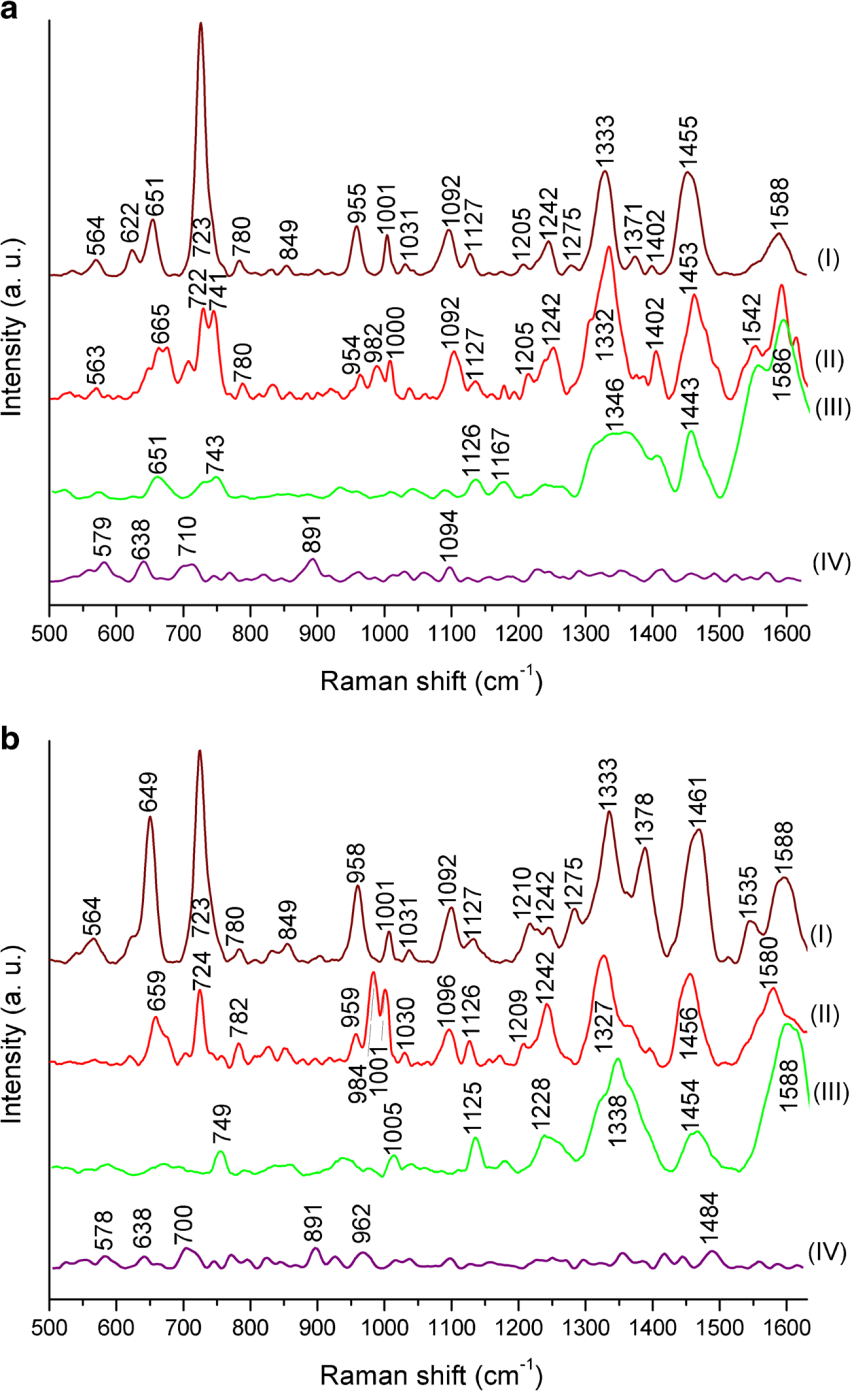
The SERS spectra of (**a**) *E. coli* and (**b**) *B. subtilis* measured with four different laser lines: (**I**) 785 nm, (**II**) 633 nm, (**III**) 532 nm, and (**IV**) 325 nm. Bacteria were cultured on LB agar medium (24 h, 37 °C). All presented spectra were averaged from 30 SERS measurements performed on Ag/steel mesh SERS substrates, baseline corrected, smoothed, and normalized

The results of SERS measurements of *E. coli* and *B. subtilis* performed with four different laser lines (785 nm, 633 nm, 532 nm, and 325 nm) and obtained on steel mesh substrate covered with Ag nanostructures are presented in Fig. 4 (the row, unprocessed spectra are shown in ESM Fig. S6). For all bacterial samples, the same conditions of cell culture (37 °C, 24 h) and sample preparation (see sections “Culture media and conditions of bacterial cultivation” and “Sample preparation for SERS measurements”) were maintained. The measurements of *E. coli* and *B. subtilis* performed on the Ag/steel substrate using 325-nm and 532-nm laser lines resulted in spectra with almost no bands in the case of 325-nm line or with only few bands in the case of 532-nm line (Fig. 4a, b (III and IV)). Furthermore, the spectra obtained with the 532-nm line were difficult to interpret, as the background fluorescence was very high and the background correction was indispensable. On the contrary, the measurements conducted with 633-nm and 785-nm laser lines (Fig. 4a, b (I and II)) gave the most satisfactory results, as the obtained SERS spectra showed a lot of bands originating from components of bacterial cell. However, after applying the 785-nm laser line, the obtained spectra showed, to some extent, a higher value of SNR than for experiments engaging the 633-nm line (see ESM Fig. S6). Additionally, the intensities of the bands in the spectra obtained with the 785-nm line were higher compared to those in the spectra recorded using the 633-nm line.

The biggest signal enhancement in SERS appears when both surface and resonance effects are combined. The resonance effect is the highest when the *λ*_max_ of localized surface plasmon resonance (LSPR) is located between *λ*_ex_ (the exciting wavelength, here at 785 nm) and *λ*_vib_ (the wavelength that is Raman scattered by the analyte, here at 833 nm, corresponding to the band at 731 cm^−1^) [52]. For silver nanostructures, the *λ*_max_ (the wavelength of maximum extinction) of LSPR is located at around 400–500 nm [53, 54]; however, it is true for monomeric colloidal suspensions and not necessarily for structures like the ones used in the SERS substrates. It has been shown for a number of metals and structures that *λ*_max_ of LSPR can be tuned over a relatively broad range by changing the spacing and morphology of the noble metal in the array [55–59]. The closer proximity to the region between *λ*_ex_ and *λ*_vib_ should result in a greater enhancement. Nonetheless, this piece of information does not explain why the SERS measurements of bacteria performed with the 633-nm laser line are of inferior quality in comparison to the measurements performed with the 785-nm laser line. Recently, this phenomenon has been explained by the existence of plasmonic dark modes, which arise from the plasmon hybridization in a set of interacting NPs [60]. These dark modes have a zero net dipole moment in contrast to dipole-active bright modes and usually do not show up in optical absorption experiments [61]. Moreover, as all absorption measurements are done for metal NPs, it would be precarious to expect that the extinction spectrum obtained from the single metal NPs will be the same as in the case of bulk SERS substrate.

In conclusion, the results presented in Fig. 4 indicate that the laser with *λ*_ex_ = 785 nm is the best for investigation of the samples of biological origin. For this reason, all of the following SERS experiments shown in this work were performed using the 785-nm laser line.

The power of laser illuminating the sample depends on the laser spot size and the magnification of the microscope, which results in the intensity of scattered light. The biological samples are generally low scattering materials and very sensitive to the radiation damage and to the local thermal decomposition.

The burning or photodegradation of biological samples, especially over a prolonged period of excitation at a high power of laser, very often results in the presence of the characteristic band in the recorded SERS spectrum at ca. 1500 cm^−1^ associated with the formation of amorphous carbon. On the other hand, the reduced laser power during measurements results in very poor quality of spectra and generate valueless information.

In ESM Fig. S7, six SERS spectra of *E. coli* (ESM Fig. S7a) and *B. subtilis* (ESM Fig. S7b) measured using different laser power levels of the 785-nm laser line are presented. Although all the spectra show bands characteristic for investigated bacteria, in the case of 0.08 mW, 0.16 mW, and 0.8 mW laser power levels, SNR was quite low. This issue was omitted when the measurements were performed with 1.5 mW, 8.0 mW, and 16.0 mW laser power levels. However, when the measurements for 8.0 mW and 16.0 mW took more than 1 min in the same spot of SERS substrate, the band of amorphous carbon appeared in the spectrum. These results indicate that high power of the laser light may cause damage of bacterial cells. For this reason the next SERS measurements were performed using a laser power of 1.5 mW.

The graph of intensity dependence on the laser power for the most prominent SERS band in the spectra of *E. coli* and *B. subtilis* is depicted in ESM Fig. S8.

### The influence of the type of culture medium

Media for the cultivation of microorganisms contain the substances, which are necessary to support the growth of microorganisms. It has been already shown that different culture media give different SERS spectra [62]. Slight differences in the composition of specific medium can result in a substantial change in the growth characteristics of microorganisms. The use of different media for the growth of the tested bacteria, *E. coli* and *B. subtilis*, affects the diversity of their composition and surface properties. Due to the fact that a SERS technique, as a method of bacterial detection and identification, is based mainly on the investigation of the cell wall and membrane composition, it seems important to examine the impact of diversified bacterial culture media on the obtained SERS results. To study this dependency, we chose the most important and popular growth media available on the market: LB agar and BHI agar for cultivation of both *E. coli* and *B. subtilis*, MEYP agar for cultivation of *B. subtilis*, and TBX agar and CCA for cultivation of *E. coli*. The short description of mentioned culture media is given in ESM Table S1.

**Fig. 5:**
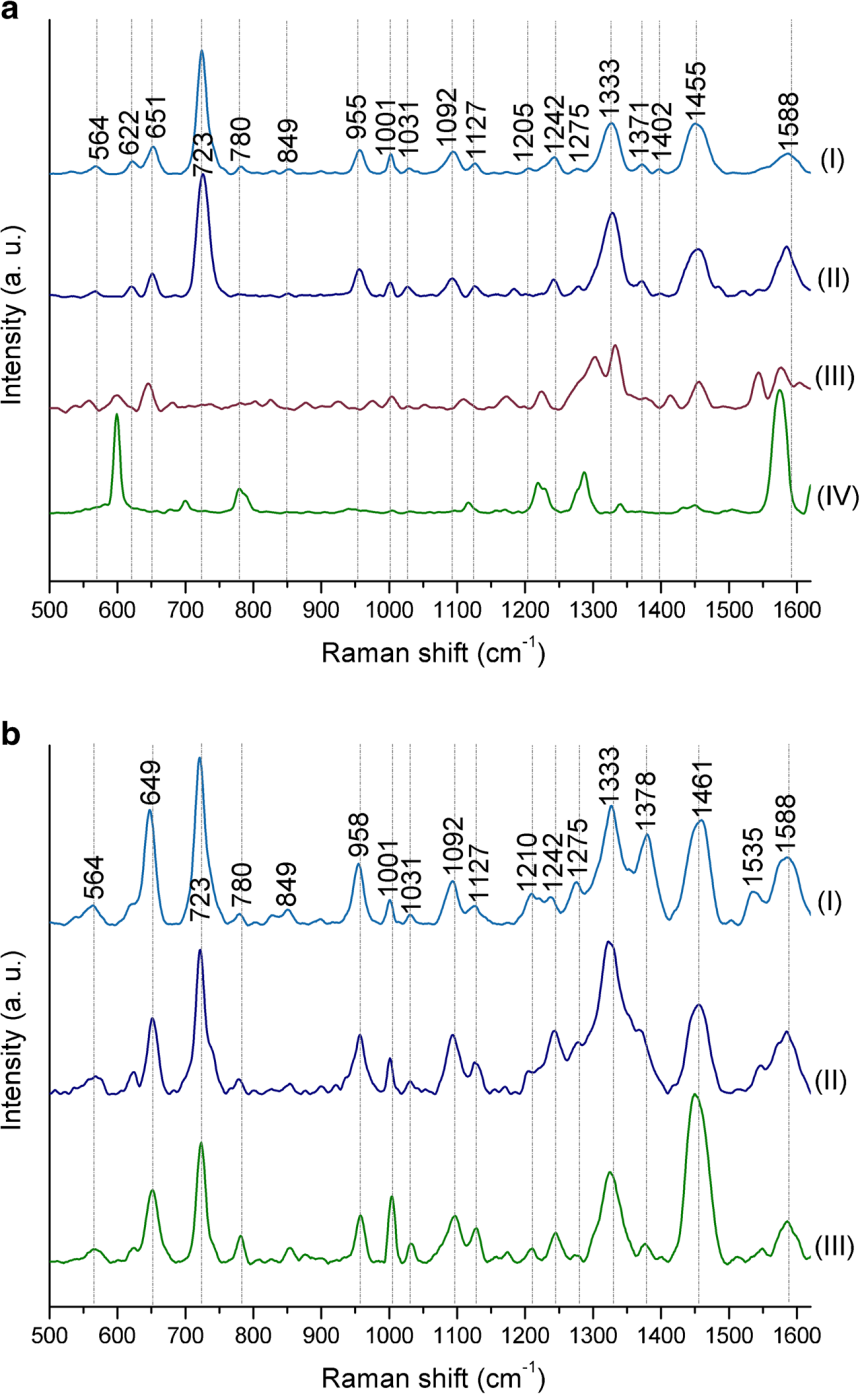
The SERS spectra of (**a**) *E. coli* cultured on (**I**) LB agar, (**II**) BHI agar, (**III**) TBX agar, and (**IV**) CCA media and (**b**) *B. subtilis* cultured on (**I**) LB agar, (**II**) BHI agar, and (**III**) MEYP agar media. Bacteria were cultured for 24 h in 37 °C. All presented spectra were averaged from 30 SERS measurements performed on Ag/steel mesh SERS substrates with the 785-nm laser line (1.5 mW), baseline corrected, smoothed, and normalized

The graphs presented in Fig. 5 show the results of SERS measurements performed on *E. coli* and *B. subtilis* grown on different culture media. The spectra of *E. coli* cultured on LB agar and BHI agar show a very high level of similarity: the band locations and intensities are almost the same. The exceptions from that rule are the bands at around 1330 cm^−1^ and 1590 cm^−1^ which exhibit higher intensities in the case of BHI agar than in LB agar. On the contrary, when measuring *E. coli* cultured on TBX agar and CCA media, one can observe completely different spectroscopic images. The bands at around 620 cm^−1^, 720 cm^−1^, 955 cm^−1^, 1092 cm^−1^, and 1242 cm^−1^, which are present for LB agar and BHI agar, are absent for TBX agar and CCA. Additionally, the bacteria grown on TBX agar show extra bands at 600 cm^−1^, 1173 cm^−1^, 1302 cm^−1^, 1420 cm^−1^, and 1546 cm^−1^, while the bacteria from CCA medium exhibit bands at around 600 cm^−1^, 700 cm^−1^, 1118 cm^−1^, 1225 cm^−1^, and 1290 cm^−1^. The mentioned bands cannot be assigned to biomolecules building bacterial cell. Their presence is connected with the chromogenic compounds which are released during bacterial growth and cover the SERS signal of bacterial cell. The only bacterial bands which can still be observed are 650 cm^−1^, 1330 cm^−1^, 1450 cm^−1^, and 1590 cm^−1^ on TBX agar medium. For CCA medium, the similarity to other spectra is connected only with the band at around 1590 cm^−1^; however, as this band has much higher intensity in comparison to other spectra, it probably comes from the overlap of two bands: one assigned to bacterial cell and second to chromogenic substrate from CCA medium, as the band at around 1590 cm^−1^, following different scientific sources, can be assigned to phenylalanine, hydroxyproline, tyrosine, C–N stretching, NH_2_ scissors, COOH antisymmetric stretching, or ring stretching of adenine/guanine.

### The influence of the time of bacterial culture

In order to define the cell culture period needed to obtain the most reliable results and the strongest Raman signal enhancement, *E. coli* and *B. subtilis* bacteria were cultured for 24 h, 48 h, and 72 h, at 37 °C, on the medium frequently used in microbiology, i.e., LB agar. This procedure is necessary, as the cell composition, structure, and amount of produced metabolites may change with time. After these periods, SERS measurements on Ag substrate were performed.

**Fig. 6:**
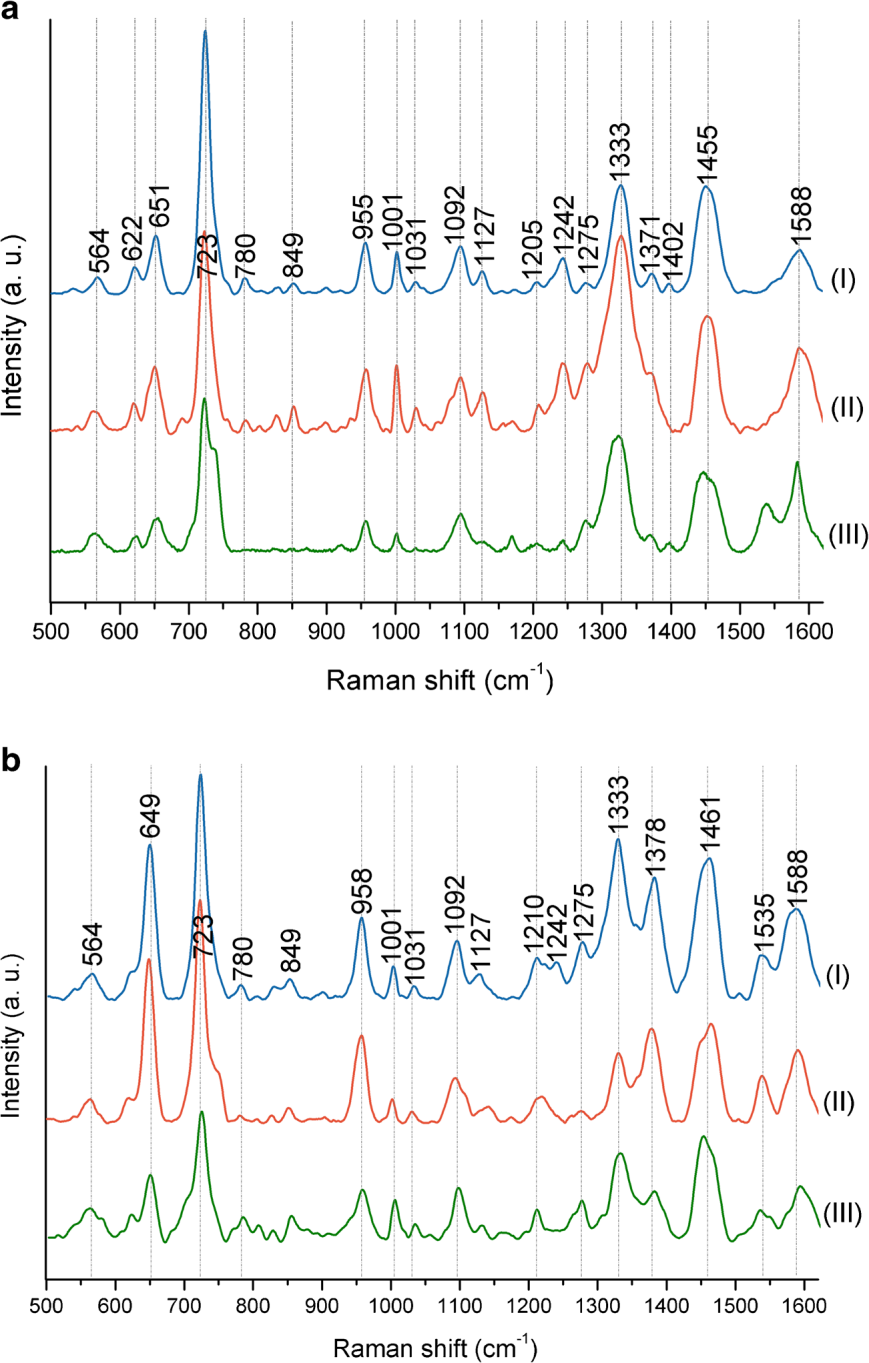
The SERS spectra of (**a**) *E. coli* and (**b**) *B. subtilis* after (**I**) 24 h, (**II**) 48 h, and (**III**) 72 h of culture on LB agar medium (37 °C). All presented spectra were averaged from 30 SERS measurements performed on Ag/steel mesh SERS substrates with the 785-nm laser line (1.5 mW), baseline corrected, and smoothed

The results are presented in Fig. 6. As one can notice, after 24 h and 48 h of culture period, the majority of SERS bands remained strongly separated from the background for both species. Additionally, on these spectra, almost all band characteristics for bacterial cells could be detected; however, for *E. coli* after 48 h of culture (Fig. 6a (II)), one can observe slightly decreased intensity of the band at around 725 cm^−1^ and increased intensity of the band at around 1330 cm^−1^. After 72 h of cell culture of *E. coli* (Fig. 6a (III)), the intensity of numerous bands dropped down (e.g., at around 780 cm^−1^, 850 cm^−1^, 1030 cm^−1^, and 1130 cm^−1^), similar to bands at 650 cm^−1^ and 725 cm^−1^ in the case of *B. subtilis* (Fig. 6b (III)). These findings are probably connected with the amount of dead bacterial cells which increases with time. As a result of cell death, the integrity of cell wall and cell membrane, which are responsible for the main contribution to the recorded SERS spectrum, decreases. Here, it should also be mentioned that while after 72 h of cell culture a high number of dead cells might lower the signal intensity, other valuable processes connected with cell death can still be studied by SERS (as long as the conditions are kept consistent within experiments).

For the above reason, it was concluded that bacteria cultured for 24–48 h are the material giving the appropriate SERS signal intensity and, thus, are suitable for further SERS measurements; however, in order to compare any results, only one cell culture time (24 h or 48 h) should be chosen.

### Influence of different bacterial treatment conditions

**Fig. 7:**
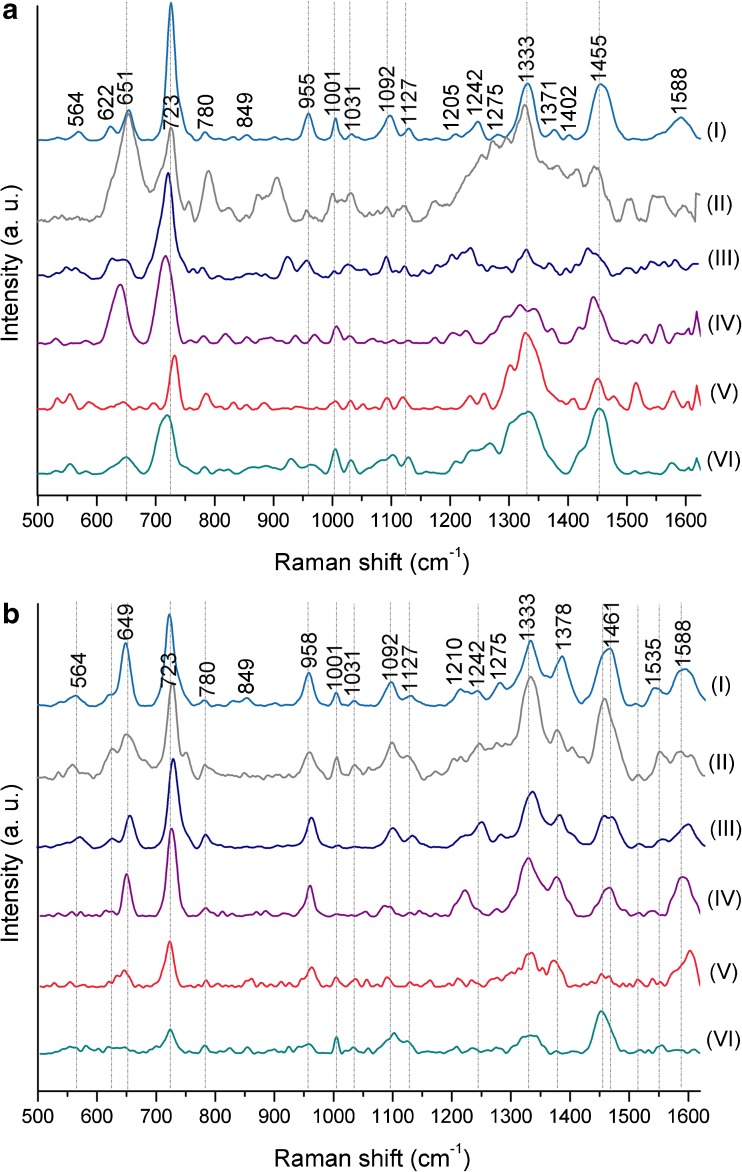
The SERS spectra of (**a**) *E. coli* and (**b**) *B. subtilis* after (**II**) suspending in 70% ethanol, (**III**) freezing in − 80 °C, (**IV**) centrifugation (13,150×*g*), (**V**) heating up to 100 °C, and (**VI**) exposition to UV light. The averaged spectrum of bacterial cells from 24-h culture in 37 °C (**I**) was given for comparison. All presented spectra were averaged from 30 SERS measurements performed on Ag/steel mesh SERS substrates with the 785-nm laser line (1.5 mW), baseline corrected, smoothed, and normalized

The easiest and most popular method of killing microbial cells is putting them into contact with ethanol solution [63], preferably 70–85% aqueous EtOH solution. The presence of water is necessary in order to penetrate the cell wall more completely. As a result, the EtOH solution fills the entire cell, coagulates all proteins, and destroys or inhibits the growth of bacteria. Water is also needed to slow evaporation and, thus, to increase surface contact time. The EtOH solution with the concentration > 90% coagulates proteins immediately and, therefore, causes the formation of a layer which protects other proteins from further coagulation. As one can notice in Fig. 7, the spectra of both *E. coli* and *B. subtilis* changed a lot after their interaction with alcohol (Fig. 7a, b (II)). Although the main bacterial bands (~ 650 cm^−1^, 720 cm^−1^, 1330 cm^−1^, and 1450 cm^−1^ for both bacterial species and ~ 960 cm^−1^, 1000 cm^−1^, and 1090 cm^−1^ for *B. subtilis*) are discernible, the other spectral regions have changed significantly, especially in the case of *E. coli*. What is also worth mentioning is the increase of the band intensity at around 650 cm^−1^.

In the next step, bacteria were exposed to low temperature (− 80 °C; see Fig. 7a, b (III)). Such treatment also causes destruction of microbial cells. This damage is connected with the fact that during the freezing process, the water is converted to ice and solutes accumulate in the residual water. Their high concentration can cause denaturation of biomolecules leading to bacterial death. To avoid the negative effects of freezing, a cryoprotectant such as glycerol is often used [64]. In the present study, bacterial cells were frozen in saline solution, without any glycerol added. As in the case of ethanol treatment, the SERS spectrum of *E. coli* has changed a lot—the intensities and shapes of various bands are different in comparison to viable bacterial cells, while the spectroscopic image of *B. subtilis* remains almost the same, with only slight changes in some band intensities.

The similar observations apply to SERS spectra obtained after centrifugation (13,150×*g*; Fig. 7a, b (IV)) which has been known to alter bacterial cell surface properties and interior structures, including DNA [65]. It was shown that centrifugation at 15,000×*g* causes significant reductions in *E. coli* viability compared to centrifugation at 5000×*g*, while a minute effect was detected on the viability of *Psychrobacter* sp. or *Staphylococcus epidermidis* [66]. It can be concluded that *E. coli* cells are extremely sensitive to centrifugation in contrast to other bacterial species. This can be noticed in SERS spectra: the differences between spectra of viable and strongly centrifuged *E. coli* are clearly discernible (a lot of bands simply disappeared after centrifugation), which is not the case for *B. subtilis* (the slight differences are limited to the region between 1040 and 1250 cm^−1^).

The high temperature also has bactericidal properties. For this reason, it is used in food production, during pasteurization process. In the case of heating bacterial cells up to 100 °C, the denaturation of all bacterial proteins occurs. These changes can be seen in the SERS spectrum of *E. coli* and *B. subtilis* (Fig. 7a, b (V))—only the bands at 720 cm^−1^ and 1330 cm^−1^ are characterized by substantial intensities.

Similar results are obtained for the microbial samples exposed to UV light. It was demonstrated that the 200–280 nm range of UV radiation, called UVC, has germicidal properties [67]. UVC light inactivates microorganisms by damaging their DNA due to the dimerization of thymine bases [68]. SERS spectra of bacteria treated with UVC light are shown in Fig. 7a, b (VI). The spectra of both species show bands with decreased intensities at around 720 cm^−1^, 1000 cm^−1^, 1090 cm^−1^, 1330 cm^−1^, and 1450 cm^−1^, while other spectroscopic features are difficult to observe.

The influence of the time spent of bacterial sample on the SERS platform has been also examined, as the toxic effect of SERS substrate covered with Ag NPs may lead to the different spectral response with time. The results of this experiment are presented and described in ESM, section 8. It has been observed that the intensity of significant majority of the bands did not change considerably with time. The one of the few changes was connected with a decrease in intensity of the band at ~ 720 cm^−1^ for both investigated bacterial species. The decrease in SERS signal intensity for different spots on the substrate has also been observed, however only after few hours (data not shown). The intention of these experiments was to show that too long exposure of bacterial cells to laser light (even low powered) and Ag NPs may have impact on their spectrum and that the spots on the SERS substrate for which the measurements are done should be changed at least few times during the experiment.

These results indicate that the changes in the intensity of SERS signal of bacteria might be due to their interaction with both SERS substrate and laser light.

### Comparison of SERS spectra of *E. coli* and *B. subtilis*

In ESM Fig. S10, we present the SERS spectra of *E. coli* and *B. subtilis*. The spectra were obtained after applying the best selected culture and measurement conditions (see Table 1), due to previously shown results (Figs. 2, 3, 4, 5, 6, and 7 and ESM Figs. S5, S7, and S9). In order to show the reproducibility of the used technique, the three randomly chosen, unprocessed SERS spectra of *E. coli* and *B. subtilis* measured on three different Ag/steel mesh SERS substrates from three different batches of LB agar medium (culture conditions, 24 h, at 37 °C) are depicted in ESM Fig. S11.

**Table 1:** Selected culture and measurement conditions

Studied conditions	Best selected conditions
Normal Raman versus SERS	In order to obtain reliable, varied, free-of-noise, and strong background fluorescence spectra of bacterial cell, SERS measurements instead of Raman measurements should be performed
Base material building the SERS substrate	The base material ought to be characterized by high surface roughness and should not give contribution to measured SERS spectrum (it must be fully covered with appropriate SERS-active metal and should not be a good Raman scatterer). As long as these conditions are met, the type of base material used to produce SERS substrate should not significantly affect the obtained SERS spectra
SERS-active metal used to produce SERS substrate	Experiments should be performed on SERS substrates covered with Ag or Ag/Au (50:50) nanostructures. The best SERS enhancement was obtained for Ag and Ag/Au alloy. The Au nanostructures also give SERS enhancement of bacterial cells; however, the signal was weaker compared to Ag and Ag/Au nanostructures
Laser line wavelength and power	The red laser lines (633 nm and 785 nm) gave the lowest background fluorescence and the most characteristic SERS spectra of bacterial cells. The chosen laser power should not cause damage of the sample: for the 785-nm line, the best laser power was 1.5 mW; however, lower laser power, e.g., 0.80 mW, is also suitable
Type of bacterial culture medium	The culture media should not change the color of the cells (because of the release of chromophore which may give strong contribution to SERS spectrum, covering the signal of bacteria), unless special conditions have to be fulfilled, e.g., the use of chromogenic agars during bacterial detection in food samples, due to International Standardization Organization (ISO) protocols. The best selected media allowing further comparison of SERS spectra of different bacterial species/strains are nonspecific media, e.g., LB agar or BHI agar
Duration of bacterial cell cultivation	The cultivation of bacterial cells should not exceed 48 h. The time of cultivation longer than 2 days may cause the decrease in band intensities in SERS spectra. On the other hand, the minimum time of 24 h is often needed to obtain perceptible bacterial colonies
Additional treatment on bacterial cells	No additional treatment should be applied during sample preparation, as it may lead to damage of bacterial cells and, as a result, cause significant changes in their SERS spectra; e.g., the centrifugal force during sample preparation, if needed, should be as low as possible, especially in the case of some bacterial species, e.g., *E. coli*
Duration of the measurement	Duration of the measurement should be as short as possible (especially for the same spot). Otherwise, the decrease of the band intensity at around 720 cm^−1^ may be observed

## Conclusions

A detailed analysis of the results obtained in this work is an essential step towards understanding the interaction of bacterium with plasmonic nanostructures and to determine which compounds of bacterial cell are visible in the SERS spectrum and why. The obtained results reveal the factors that have a significant impact on the SERS spectra of bacterial cells. This is a very important issue from an analytical, especially qualitative, point of view. These studies progress the current knowledge of biospectroscopy and may start the introduction of SERS-based bacterial cell identification technique as a uniform method of analysis in every field in which detection and identification of bacteria are important. It should be noted that knowledge of bacterial species present in drinking water, air, food and body fluids can be extremely important and, in some cases, may even save human’s life, e.g., when the bacteria detected in the sample are pathogenic and can potentially cause death of the consumer/patient. Quick identification would allow for taking a proper action to minimize the risk, e.g., by starting appropriate pharmacological therapy in the case of patients with bacterial infections. The development of this field of science can lead, in the future, to the development of a new and rapid diagnostic method used in hospitals or analytical and microbiological laboratories.

This work can be treated as the protocol for appropriate preparation of SERS experiment of bacteria which may lead to standardization of worldwide obtained results.

## Electronic supplementary material

ESM 1 (PDF 11.4 kb)
