# Supplementary material for: Sources of variability in SERS spectra of bacteria: comprehensive analysis of interactions between selected bacteria and plasmonic nanostructures
Source: Anal Bioanal Chem. 2019 Mar 4;411(10):2001–17. doi: 10.1007/s00216-019-01609-4 (PMC6458985; doi:10.1007/s00216-019-01609-4)
Supplement: Supplementary file 1 — (PDF 11.4 kb) [file 216_2019_1609_MOESM1_ESM.pdf]

**Analytical and Bioanalytical Chemistry**

**Electronic Supplementary Material**

# Sources of variability in SERS spectra of bacteria: comprehensive analysis of interactions between selected bacteria and plasmonic nanostructures

Evelin Witkowska, Krzysztof Niciński, Dorota Korsak, Tomasz Szymborski,

Agnieszka Kamińska

## 1. Introduction

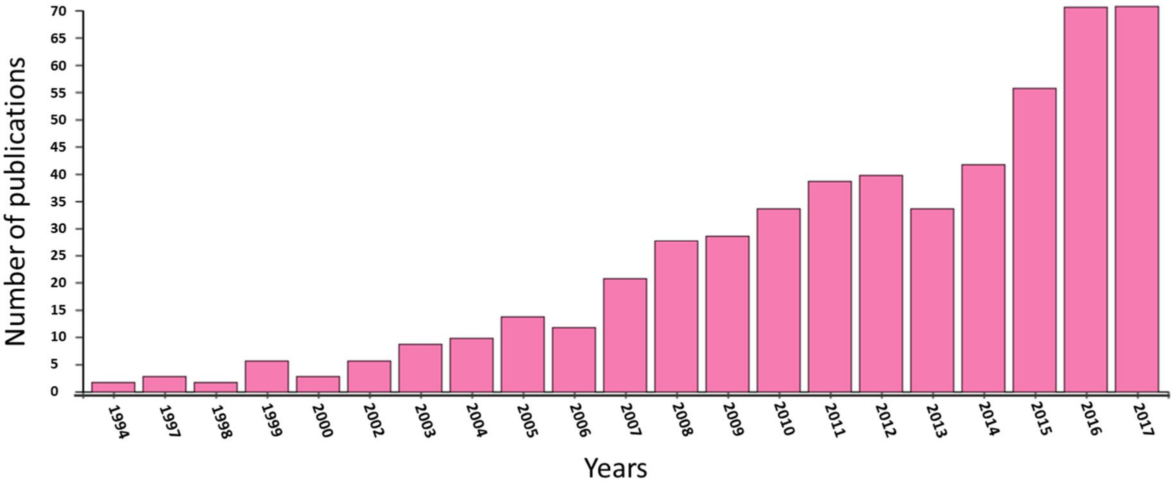


**Fig. S1** Growing popularity of the SERS technique in bacteria identification, detection or differentiation. The plot shows data from Web of Science for the topics: "SERS" and "bacteria"

## 2. Characterization of the surfaces of used SERS substrates

**a)**

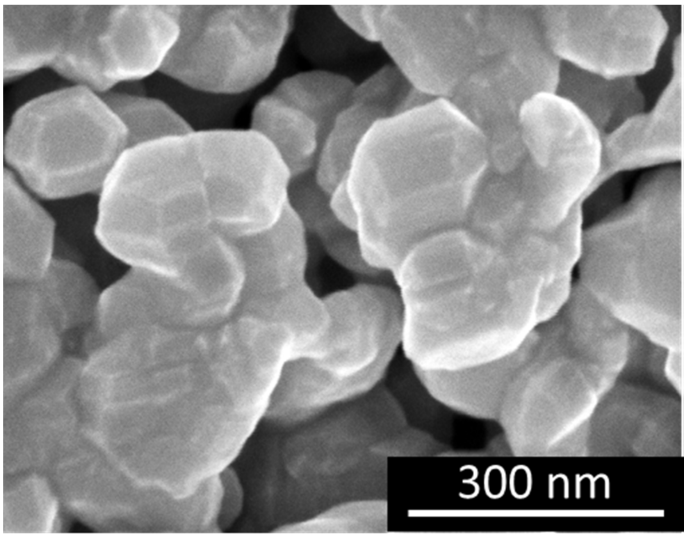


The nanostructures building the surface of Ag discs have a form of blocks with many edges. Each block is an aggregate composed of few subunits, with the diameter of 50-100 nm. Between these blocks the numerous cavities can be observed.

**b)**

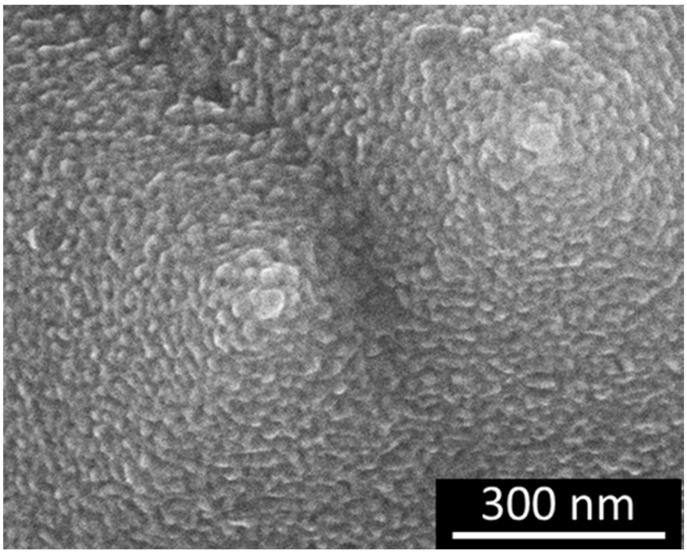


The Ag/Si SERS substrate consists of pyramids which are covered with nanostructures in a form of overlapping metal 'husks'. Each 'husk' has a diameter of 30-40 nm.

**c)**

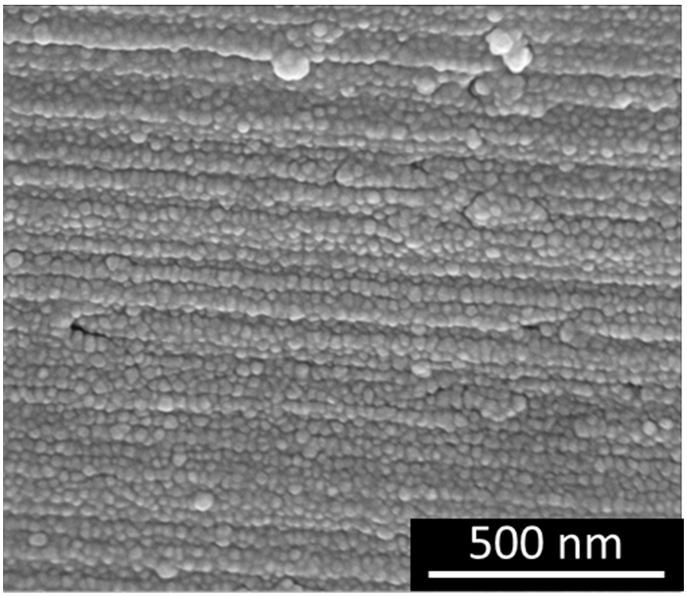


The wires which are the component of Ag/steel SERS substrate are covered with hemispheres of the diameter of 15-30 nm. These hemispheres are arranged into rows (along the long axis of the wire).

**Fig. S2** SEM images of thee different SERS substrates: (a) Silicon wafer sputtered with 10 nm of silver (b) electrochemically roughened silver disc, and (c) steel mesh sputteed with 50 nm of silver

## 3. Pristine SERS substrates

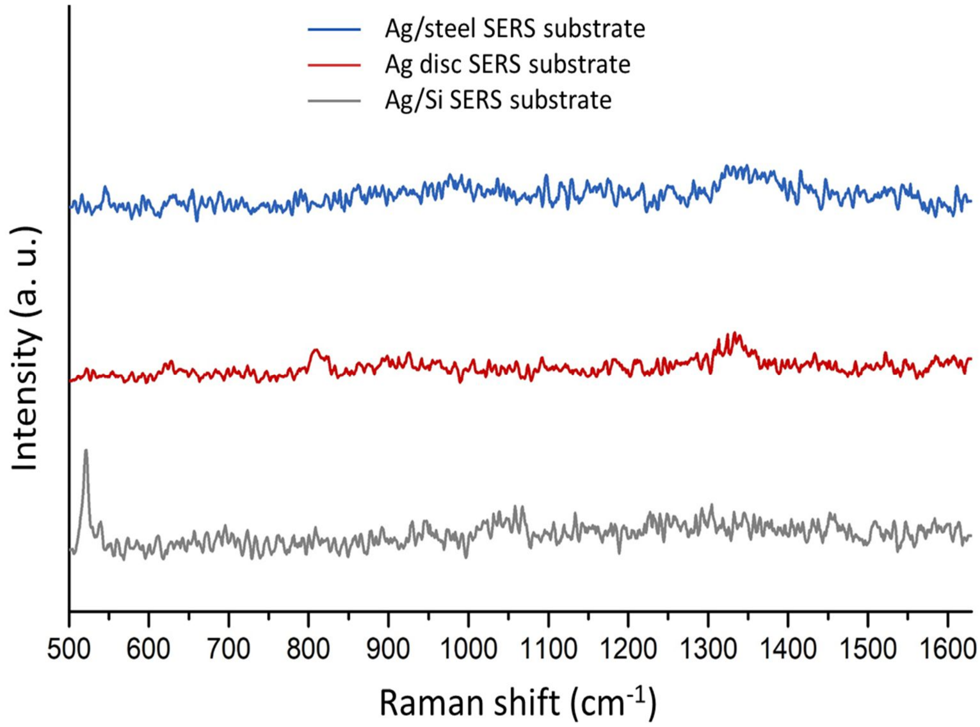


**Fig. S3** Raman spectra of pristine SERS substrtaes used during SERS measurements of bacterial cells. Presented spectra were avaraged from 10 measurements in different spots of the SERS substrate, baseline corrected and smoothed

## 4. Measured parameters

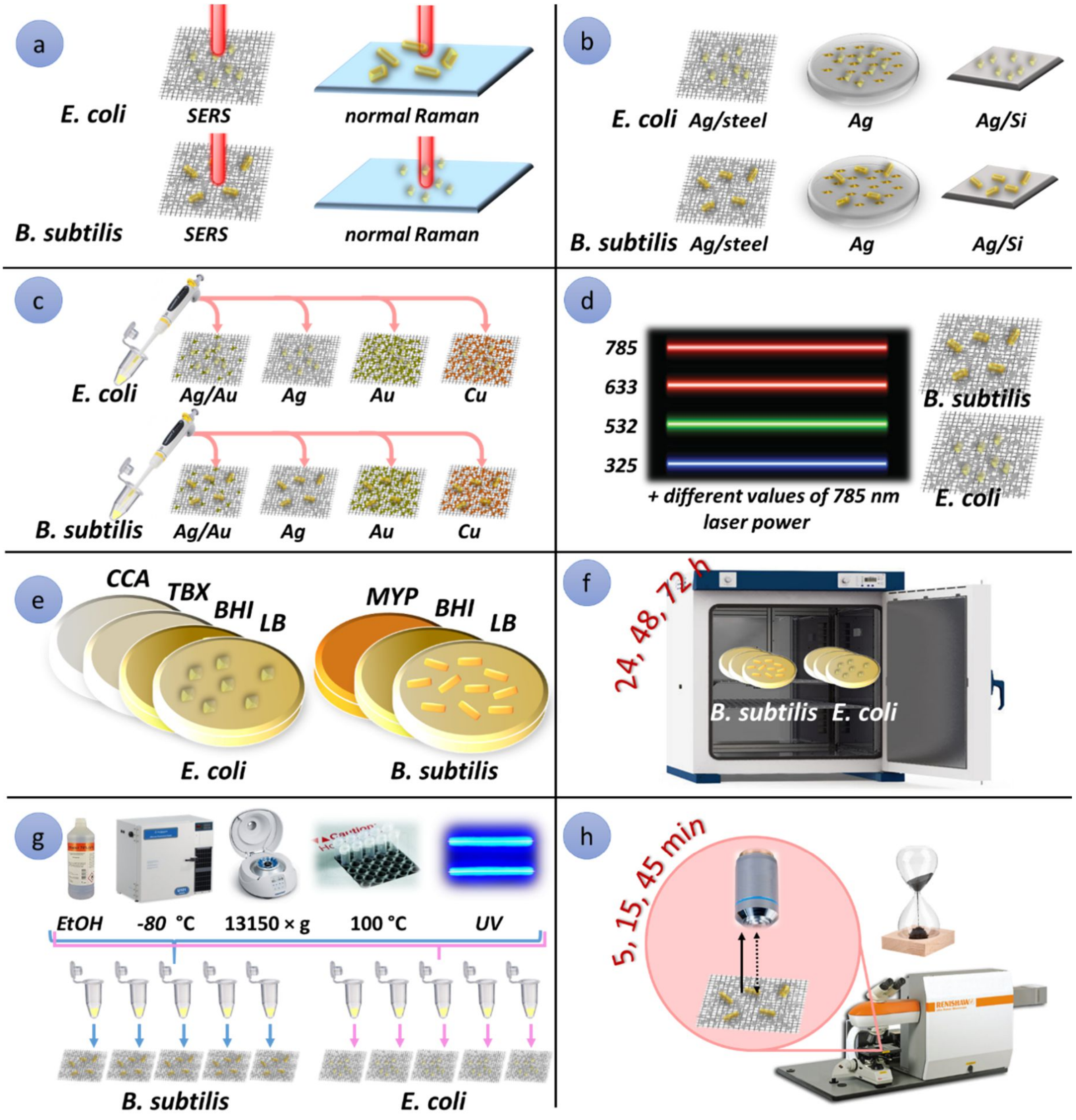


**Fig. S4** Scheme of all experimental conditions: (a) type of SERS substrates, (b) presence/absence of SERS substrate, (c) type of SERS-active metal/-s used to produce SERS substrates, (d) laser line wavelength and laser power, (e) type of culture medium, (f) time of bacteria culture, (g) different bacteria treatment, and (h) time spent on the SERS platform

## 5. Raman vs SERS

(a)

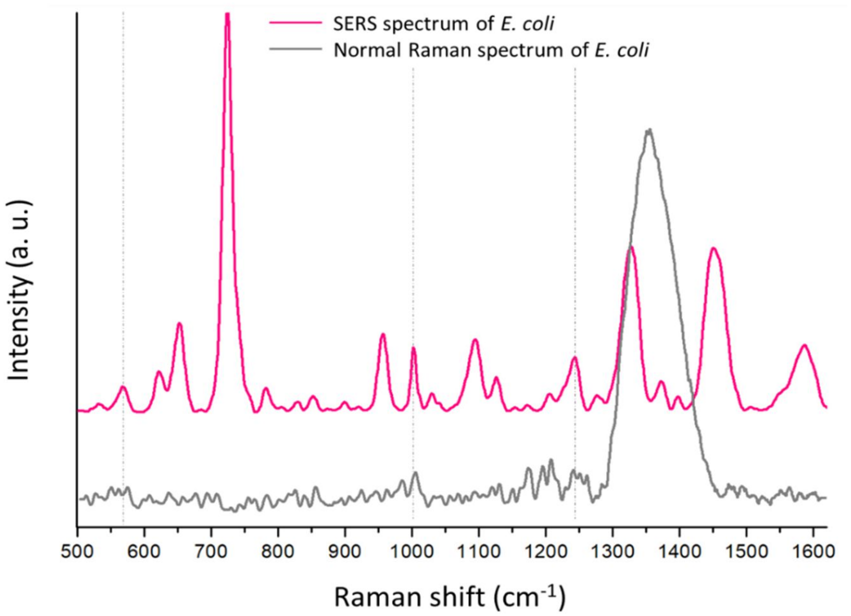


(b)

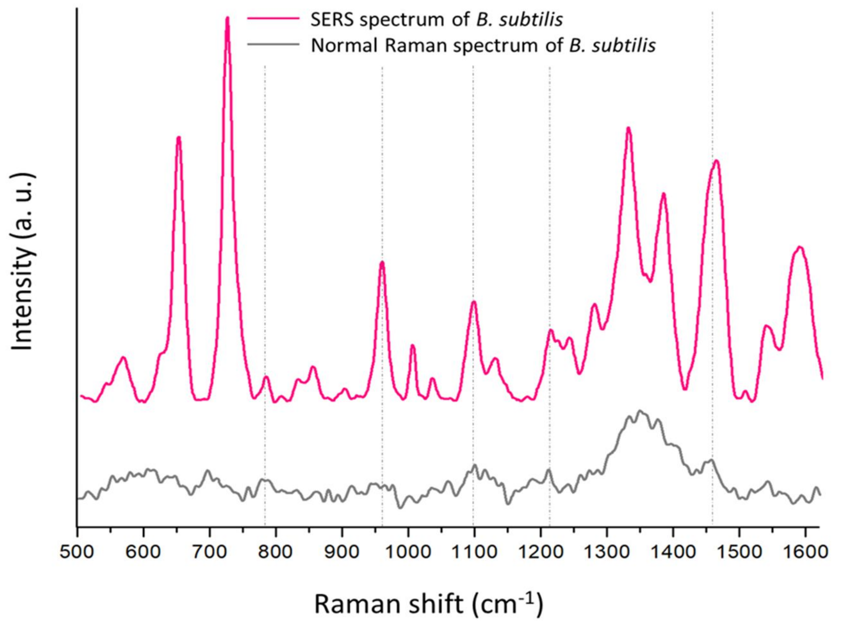


**Fig. S5** The averaged SERS (dark pink) and normal Raman (grey) spectra of (a) *E. coli* and (b) *B. subtilis* cultured on LB medium (24 h, 37 °C). The band at ~1360 $cm^{-1}$ in the Raman spectrum of *E. coli* corresponds to the strong background fluorescence. All presented spectra were averaged from 30 measurements performed with 785 nm laser line (1.5 mW). The SERS spectra were obtained on Ag/steel mesh SERS substrates. All spectra were baseline corrected, smoothed and normalized

## 6. Influence of the laser line

The excitation wavelength is the critical factor in SERS mechanism, which influences the type of received information (different cell components), the level of interference by fluorescence and scattering sensitivity and spatial resolution. In biological studies the near-IR, visible or UV wavelengths might be applied. UV excitation enables the resonance Raman scattering of many biological molecules, including protein residues and DNA bases. At UV excitation the background from florescence is relatively poor and the photodegradation of samples is eliminated but on the other side the SERS enhancement factor is considerably weaker than in the visible region (for common Ag and Au nanostructures).

Zeiri and Efrima[1] recorded SERS spectra of *E.coli* at 244 nm in the presence of silver NPs. They achieved very week bands in bacterial spectrum assigned mainly to aromatic amino acids and nucleic acids. The same research group[2] used 514.5 nm excitation line for SERS measurements of bacteria mixed with borohydride - reduced silver colloid. Surprisingly, they claimed, that the SERS spectra of four different bacteria: *Escherichia coli*, *Acinetobacter calcoaceticus*, *Pseudomonas aeruginosa*, *and Bacillus megaterium* are very similar. They assigned these similar spectral features to flavin derivatives present in all cell walls. Sengupta et al.[3] achieved at the same excitation line the comparable SERS spectrum for *E. coli* which was also dominated by flavin adenine dinucleotide (FAD) and flavin adenine mononucleotide (FMN) spectral fingerprints. Jarvis and Goodacre[4] have also shown that SERS spectra of *E. coli* and *B. subtilis* acquired with 532 nm excitation line onto borohydride reduced silver colloid are visually hardly to differentiate.

In summary, several research groups[5-6] that have investigated the SERS spectra of both Gram-positive and Gram-negative bacteria at 532 or 514 nm usually on gold and silver

colloids obtained bacterial SERS spectra dominated by the bands assigned to flavin derivatives, mainly FAD, which have the maximum of adsorption at about 370 and 440 nm.

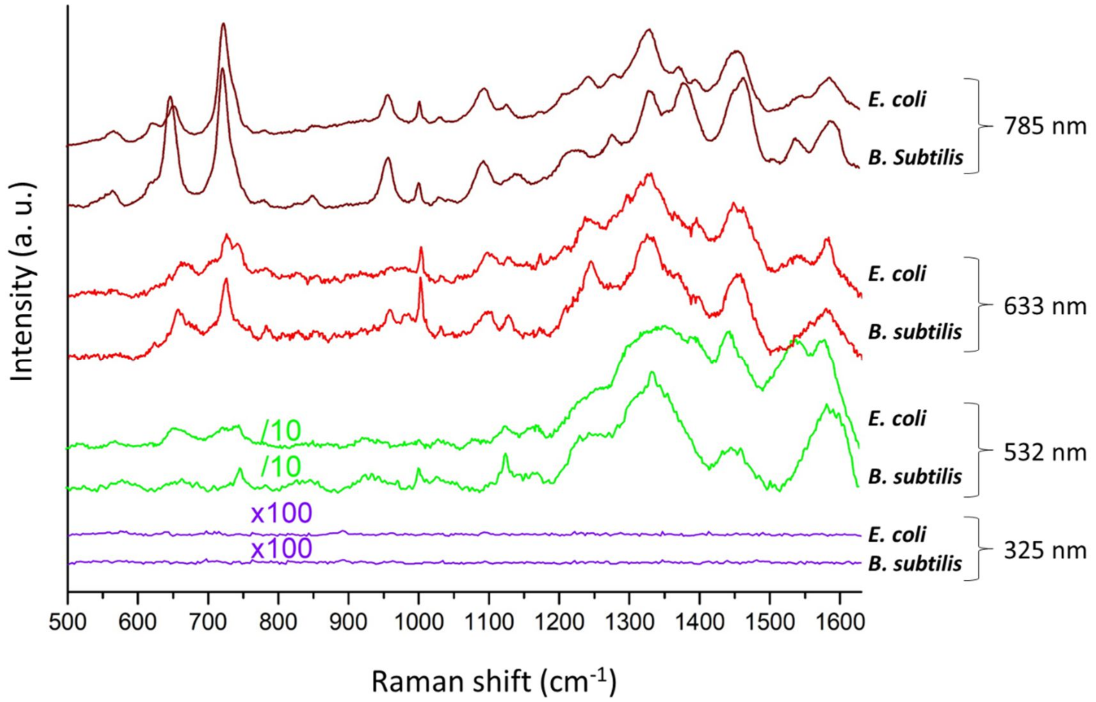


**Fig. S6** The SERS spectra of *E. coli* and *B. subtilis* measured with four different laser lines: 785 nm, 633 nm, 532 nm, and 325 nm. Bacteria were cultured on LB medium (24 h, 37 °C). All presented spectra were averaged from 30 SERS measurements performed on Ag/steel mesh SERS substrates

## 7. Influence of the laser power

(a)

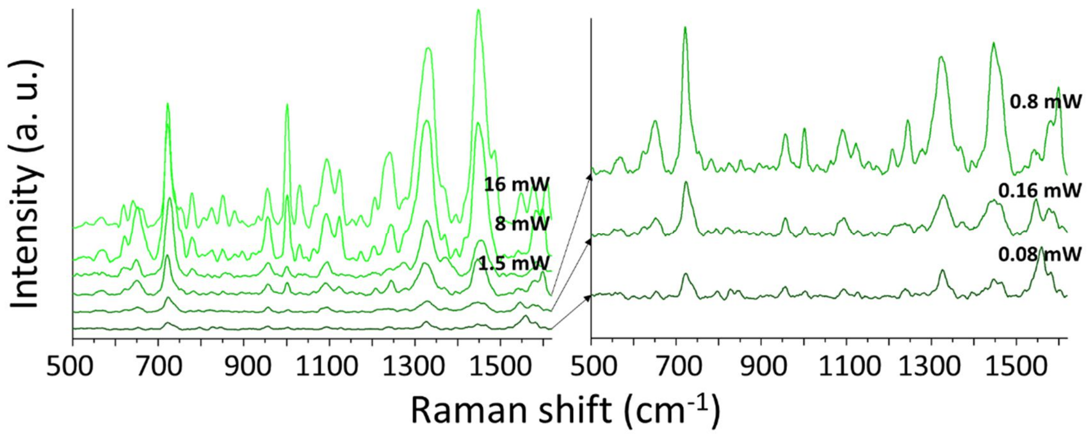


(b)

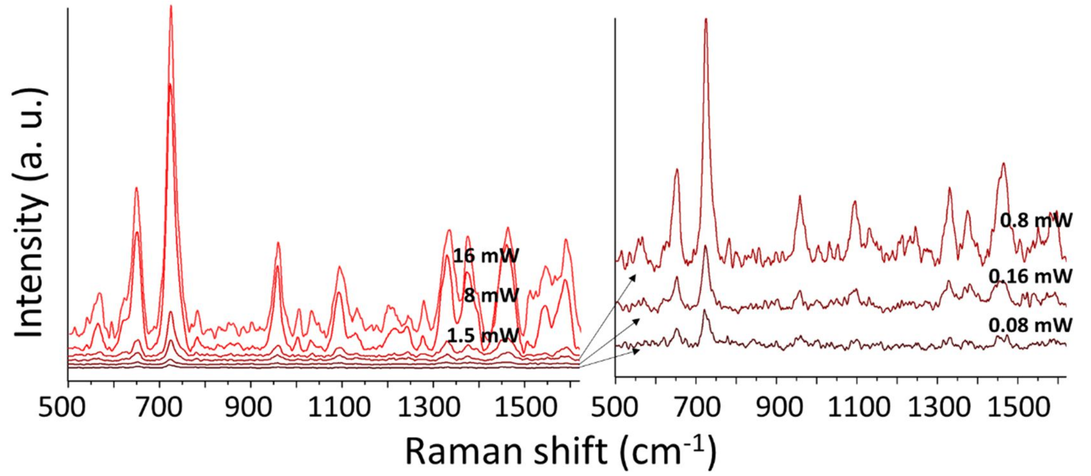


**Fig. S7** The SERS spectra of (a) *E. coli* and (b) *B. subtilis* measured with six different powers of 785 nm laser line: 0.08, 0.16, 0.8, 1.5, 8.0, 16.0 mW. Bacteria were cultured on LB medium (24 h, 37 °C). All presented spectra were averaged from 30 SERS measurements performed on Ag/steel mesh SERS substrates, baseline corrected, and smoothed

(a)

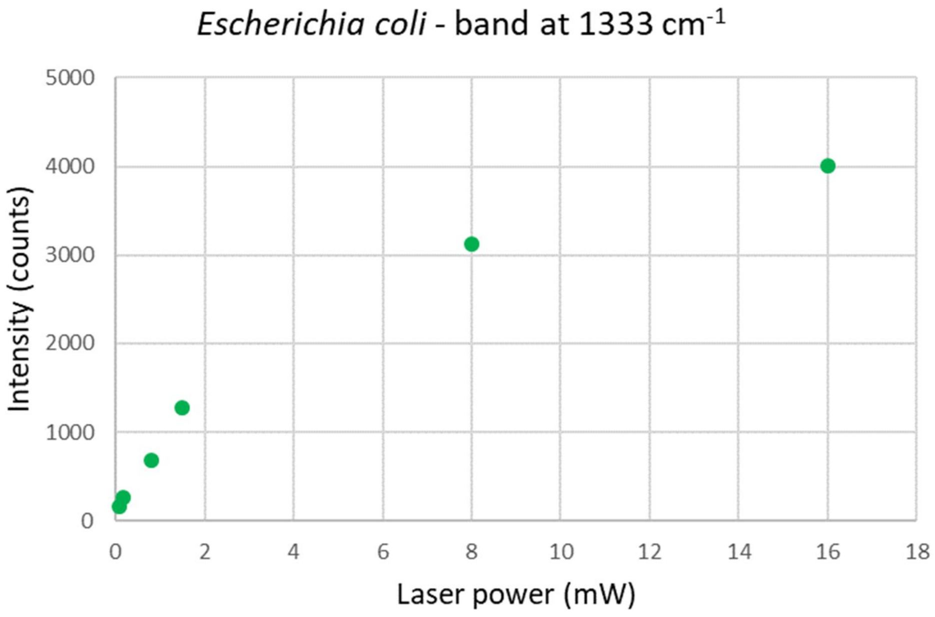


(b)

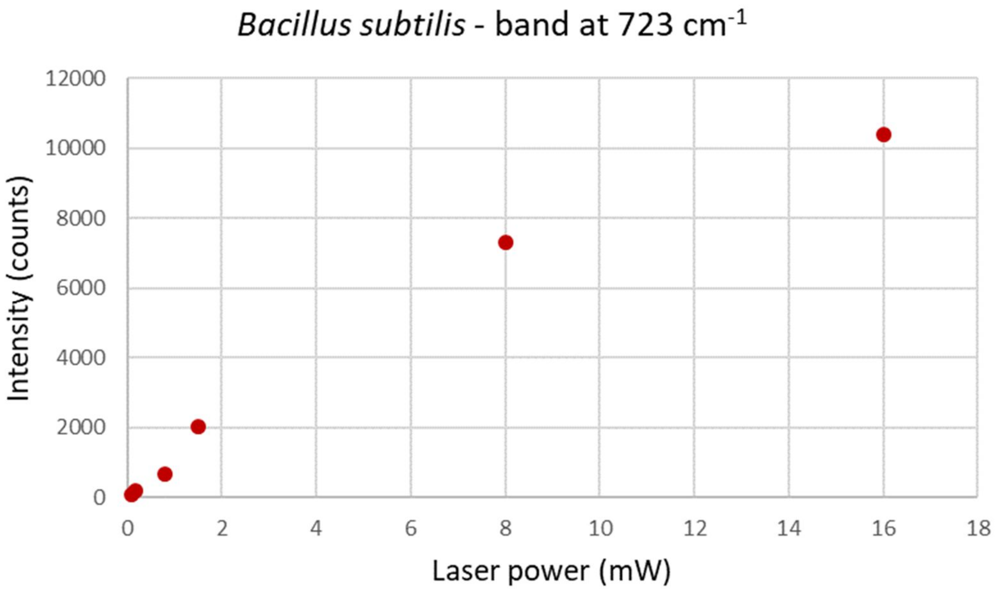


**Fig. S8** Graph of intensity dependence on the laser power for the SERS band at (a) 1333 cm$^{-1}$ in the spectrum of *E. coli* and (b) 723 cm$^{-1}$ in the spectrum of *B. subtilis*. Measurements were performed with six different powers of 785 nm laser line: 0.08, 0.16, 0.8, 1.5, 8.0 and 16.0 mW. Bacteria were cultured on LB medium (24 h, 37 °C). Each point was averaged from 30 SERS measurements performed on Ag/steel mesh SERS substrates (after baseline correction and smoothing of SERS spectra)

## 8. Influence of the time spent on the SERS platform

The time which the sample spends on the SERS substrate during measurements is also not irrelevant for its SERS spectrum. Having placed the bacterial sample on the SERS platform, the measurements were taken after 5, 15 and 45 minutes from the same spot (the time of 5 minutes for first measurement was needed in order to let the sample dry). The results without spectra normalization are presented in Fig. S8. As one can see, the intensity of the band at ~720 $cm^{-1}$ is decreasing with time for both, *E. coli* and *B. subtilis*. The same observations concerns the band at 620 $cm^{-1}$ in the spectrum of *E. coli* and at about 620 and 650 $cm^{-1}$ in the spectrum of *B. subtilis*. Nevertheless, in the case of *E. coli*, we may also observe the increase in intensity in few bands with time, e. g. at 1455 $cm^{-1}$. On the contrary, the same band (at around

1460 $cm^{-1}$) decreases in the spectrum of *B. subtilis*. Besides the given examples it seems that the intensity of most of the bands does not change considerably.

From these experiments we can conclude that the SERS measurements of bacterial cells should be performed as quick as possible in order to avoid the intensity decrease of the most important bacterial band at ~720 $cm^{-1}$. However, if this condition is not meet, the amount of time spent by bacterial sample on the SERS substrate (up to 45 minutes) does not affect the obtained spectra significantly.

(a)

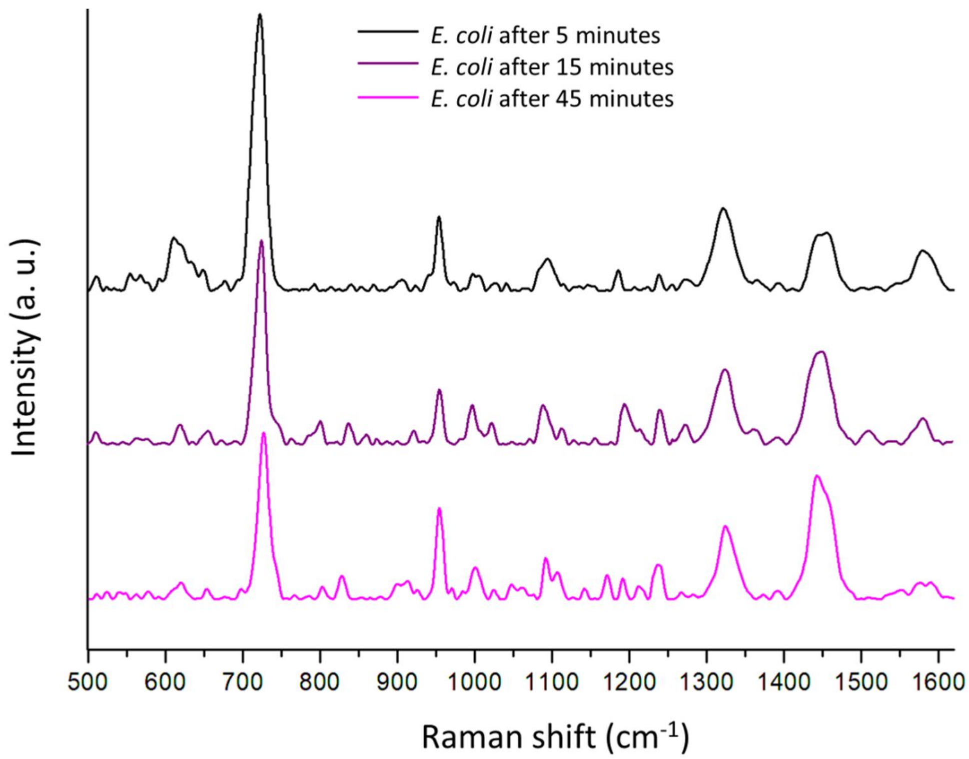


(b)

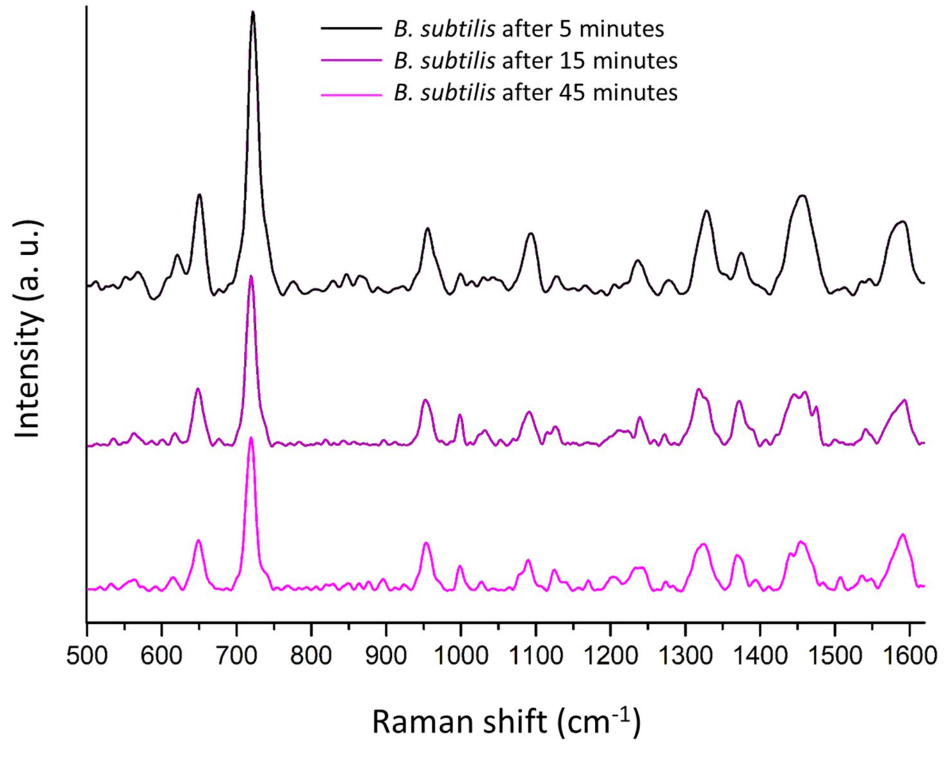


**Fig. S9** The SERS spectra of (a) *E. coli* and (b) *B. subtilis* after 5 (dark violet), 15 (violet) and 45 minutes (light violet) from the sample placement on the SERS substrate. Bacteria were cultured on LB medium (24 h, 37 °C). All measurements were performed on Ag/steel mesh SERS substrates with 785 nm laser line (1.5 mW), baseline corrected, and smoothed

## 9. Culture media used in the experiment

**Table S1** Description of culture media used for cultivation of *E. coli* and *B. subtilis*

| Bacterial growth medium | Specification/description |
|---|---|
| **Lysogeny broth (LB)** 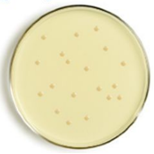 | it is a nutrient-rich, non-specific type of medium. Peptides and peptone are provided mainly by tryptone, while vitamins and trace elements - by yeast extract. Sodium ions crucial for transport and osmotic balance are provided by NaCl. |
| **Brain Heart Infusion (BHI)** 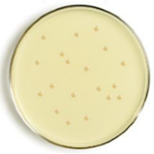 | it is a nutrient-rich, non-specific medium for the cultivation of a variety of fastidious and nonfastidious aerobic and anaerobic microorganisms. BHI typically contains infusion of beef or pig heart and calf brain, which is the source of amino acids. Other ingredients: NaCl, $Na_2HPO_4$ and glucose. |
| **Tryptone Bile X-Glucuronide (TBX)** 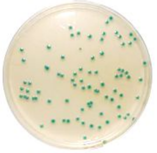 | it is a specific medium used for detection and enumeration of *E.coli* in foodstuffs, animal food and water. The chromophore released in TBX medium (X-glucuronide) is insoluble and accumulates within the cell, what makes bacteria colonies easy to identify. The X-glucuronide is targeted by glucuronidase enzyme present in 97 % of *E. coli* strains. The enzyme splits the bond between chromophore and glucuronide. Chromophore is released and as a result *E. coli* colonies are blue/green. |
| **Chromogenic Coliform Agar (CCA)** 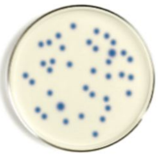 | it is a specific medium, which uses two enzyme substrates to differentiate between *E. coli* and other coliforms. One chromogenic substrate is cleaved by glucuronidase, while the second - by galactosidase, enzyme produced by the majority of coliforms. As a result *E. coli* colonies are purple, as they are able to cleave both substrates, while Coliform colonies are pink as they cleave only galactosidase. |
| **Mannitol Egg Yolk Polymyxin (MYP)** 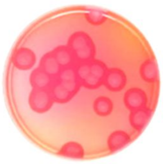 | it is a medium used with supplements for the selective and differential enumeration of *Bacillus cereus* from foods. Beef extract and peptone provide nitrogen, vitamin, and carbon, while D-mannitol is the source of carbohydrates. The fermentation process of mannitol is detected by the pH indicator - phenol red. *B. cereus* is typically mannitol-negative and it growth results in pink colonies. Supplementing with egg yolk emulsion provides lecithin, which is hydrolyzed by lecithinase, produced by *B. cereus*. As a result *B. cereus* forms a zone of white precipitation around colonies. The growth of most other bacteria is inhibited by Polymyxin B. |

## 10. Final results

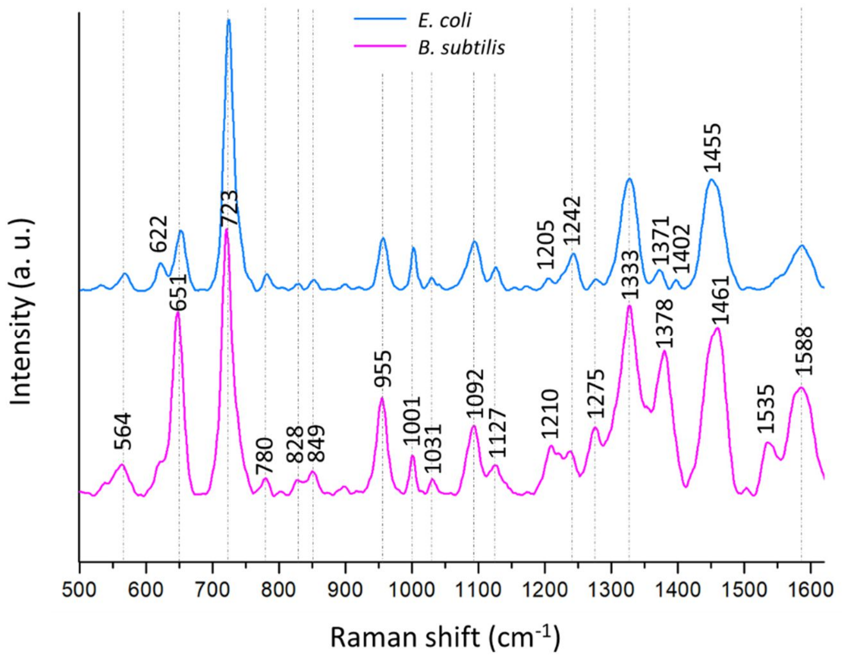


**Fig. S10** The averaged spectra of *E. coli* (blue) and *B. subtilis* (pink) from 24 h culture in 37 °C on LB agar medium. Presented spectra were averaged from 30 SERS measurements performed on Ag/steel mesh SERS substrates with 785 nm laser line (1.5 mW), baseline corrected, smoothed, and normalized

(a) (b)

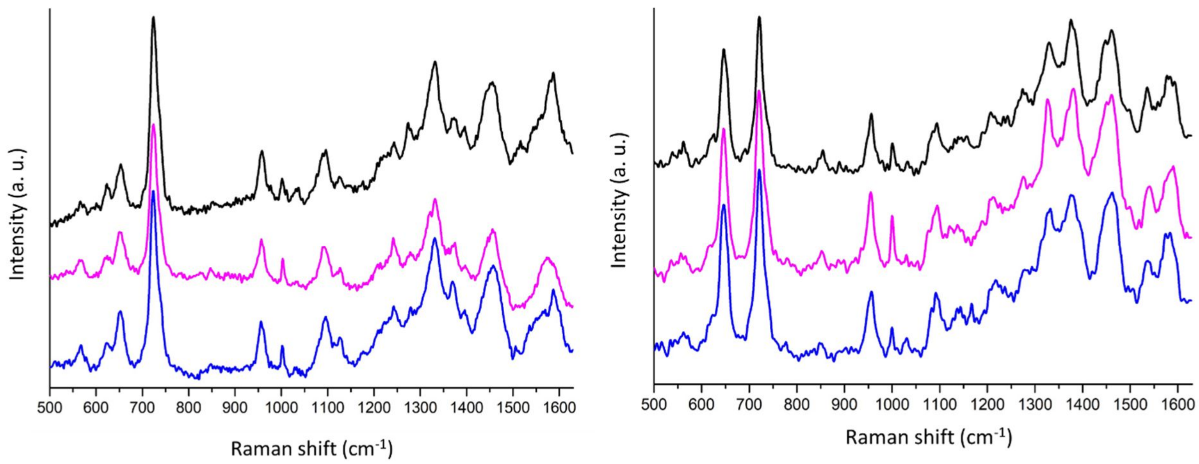


**Fig. S11** Comparison of three single, unprocessed SERS spectra of *E. coli* (a) and *B. subtilis* (b) from three different Ag/steel mesh SERS substrates and batches of LB medium (culture conditions: 24 h, at 37 °C). All measurements were performed with 785 nm laser line (1.5 mW)

## References


1. Zeiri, L.; Efrima, S., Surface-enhanced Raman spectroscopy of bacteria: the effect of excitation wavelength and chemical modification of the colloidal milieu. Journal of Raman Spectroscopy 2005, *36* (6-7), 667-675.

2. Zeiri, L.; Bronk, B. V.; Shabtai, Y.; Czégé, J.; Efrima, S., Silver metal induced surface enhanced Raman of bacteria. Colloids and Surfaces A: Physicochemical and Engineering Aspects 2002, 208 (1–3), 357-362.

3. Sengupta, A.; Mujacic, M.; Davis, E. J., Detection of bacteria by surface-enhanced Raman spectroscopy. Anal. Bioanal. Chem. 2006, *386* (5), 1379-1386.

4. Jarvis, R. M.; Goodacre, R., Characterisation and identification of bacteria using SERS. Chem Soc Rev 2008, 37 (5), 931-6.

5. Smith, G. B.; Oshima, K. H. Development of a Laser-Based Detection System for Water-Borne Pathogens; New-Mexico Water Resources Research Institute: 2004.

6. Kahraman, M.; Yazici, M. M.; Sahin, F.; Culha, M., Experimental parameters influencing surface-enhanced Raman scattering of bacteria. Journal of biomedical optics 2007, 12 (5), 054015.
